# Genome-wide identification of ubiquitin proteasome subunits as superior reference genes for transcript normalization during receptacle development in strawberry cultivars

**DOI:** 10.1186/s12864-021-07393-9

**Published:** 2021-01-28

**Authors:** Jianqing Chen, Jinyu Zhou, Yanhong Hong, Zekun Li, Xiangyu Cheng, Aiying Zheng, Yilin Zhang, Juanjuan Song, Guifeng Xie, Changmei Chen, Meng Yuan, Tengyun Wang, Qingxi Chen

**Affiliations:** 1 College of Horticulture, Fujian Agriculture and Forestry University, No. 15 Shangxiadian Road, Fuzhou, 350002 China; 2 FAFU-UCR Joint Center for Horticultural Biology and Metabolomics, Haixia Institute of Science and Technology, Fujian Agriculture and Forestry University, Fuzhou, 350002 China

**Keywords:** Reference gene, Strawberry, Receptacle development, Ubiquitin 26S proteasome system

## Abstract

**Background:**

Gene transcripts that show invariant abundance during development are ideal as reference genes (RGs) for accurate gene expression analyses, such as RNA blot analysis and reverse transcription–quantitative real time PCR (RT-qPCR) analyses. In a genome-wide analysis, we selected three “Commonly used” housekeeping genes (HKGs), fifteen “Traditional” HKGs, and nine novel genes as candidate RGs based on 80 publicly available transcriptome libraries that include data for receptacle development in eight strawberry cultivars.

**Results:**

The results of the multifaceted assessment consistently revealed that expression of the novel RGs showed greater stability compared with that of the “Commonly used” and “Traditional” HKGs in transcriptome and RT-qPCR analyses. Notably, the majority of stably expressed genes were associated with the ubiquitin proteasome system. Among these, two 26 s proteasome subunits, *RPT6A* and *RPN5A,* showed superior expression stability and abundance, and are recommended as the optimal RGs combination for normalization of gene expression during strawberry receptacle development.

**Conclusion:**

These findings provide additional useful and reliable RGs as resources for the accurate study of gene expression during receptacle development in strawberry cultivars.

**Supplementary Information:**

The online version contains supplementary material available at 10.1186/s12864-021-07393-9.

## Background

The cultivated octaploid strawberry (*Fragaria* × *ananassa*) is an important fruit crop grown worldwide. The wild diploid strawberry (*Fragaria vesca*) has emerged as a model system for strawberry made possible by the availability of a draft genome sequence (~ 240 Mb) and its relative transformability [1]. In botanical terms, the fruit of strawberry is an aggregate fruit composed of multiple achenes on the surface of the juicy flesh, which is accessory tissue developed from the enlarged receptacle (Fig. S1). The process of strawberry fruit development is divided into the early phase dominated by growth, and the ripening phase when the achenes enter dormancy accompanied by dramatic developmental changes in the receptacle, such as color changes, softening, and flavor development. The regulatory mechanism of fruit development is of considerable interest to plant scientists and breeders. In particular, elucidation of the molecular events involved in fruit development is required. Quantification of gene expression levels is crucial to unravel this complex regulatory network. Reverse transcription–quantitative real time PCR (RT-qPCR) is a favored approach used for quantification of gene expression on account of its specificity, accuracy, and reproducibility. Accurate normalization is fundamental for reliable analysis of RT-qPCR data. Therefore, this technology requires stably expressed reference genes (RGs) for expression normalization of target genes. Failure to use an appropriate RG may lead to biased gene expression profiles and low reproducibility.

Traditional housekeeping genes (HKGs) are used commonly as RGs on the basis of their essential cellular roles and therefore are thought to be stably expressed. To date, the RG transcripts most frequently used for RT-qPCR in strawberry fruit studies include three traditional HKGs that encode the 26–18S rRNA intergenic spacer [2, 3], Actin [4, 5], and glyceraldehyde-3-phosphate dehydrogenase (GAPDH) [6, 7]. Unfortunately, traditional HKGs, including the four HKGs used in strawberry fruit studies, are utilized generally without validation of their stability and based on the supposition that the genes are expressed at constant levels under all conditions. Increasing evidences question the reliability of traditional HKGs, which can be subject to considerable variation under certain conditions, including different developmental stages [8]. For instance, traditional HKGs analyzed from a developmental series of *Arabidopsis* seed and pollen samples show highly variable expression [9]. Therefore, it is essential to evaluate appropriate RGs for the experimental system under study. For this purpose, several research groups have developed software, such as geNorm [10], BestKeeper [11], NormFinder [12], and Delta CT [13], which are commonly used for statistical analyses and selection of the most stably expressed RGs. In previous researches, a few members of traditional HKGs as candidate RGs were assessed in studies of strawberry fruit ripening, of which *FaRIB413* (*26–18S rRNA*), *FaACTIN*, *FaHISTH4*, *FaDBP* and *FaUBQ11* were recommended as appropriate RGs [14–17]. Unfortunately, these results were restricted in scope and rationalization to selection of the candidate genes evaluated.

Transcriptomic analyses are extensively used in investigations of complex molecular processes in plants. Deep RNA sequencing (RNA-seq) as a global evaluation technique provides a representative snapshot of a transcriptome given its globality, high resolution, and sensitivity. One strategy is to mine RNA-seq data sets for identification of the optimal RGs that are stably expressed over a diverse set of conditions. This approach has been successfully employed in several plant species, such as *Arabidopsis* [9], rice [18], and soybean [19]. Previously, Clancy et al. (2013) have identified a set of strawberry (*Fragaria* spp.) constitutively expressed RGs during strawberry fruit ripening by merging digital gene expression data with expression profiling; among these, *FaCHP1* and *FaENP1* were recommended as appropriate RGs [20]. However, this result were restricted in the statistical limitations of the study due to the small sample size. The extensive RNA-seq data sets previously generated for stages of receptacle development in strawberry provide valuable resources for screening of the optimal RGs across receptacle developmental stages [21–24].

In this study, we selected 3 “Commonly used” HKGs, 15 “Traditional” HKGs, and 9 novel genes as candidate RGs based on genome-wide and available RNA-seq data, which were assessed during receptacle development in nine independent experiments from eight strawberry cultivars. The results revealed a tendency for all novel RGs to show greater expression stability, compared with that of the “commonly used” and “traditional” HKGs, in transcriptome and RT-qPCR analyses. The genes *RPT6* and *RPN5A,* subunits of ubiquitin proteasome, are recommended as the optimal combination of RGs in strawberry receptacle development. These findings provide additional useful and reliable RGs as resources for the accurate study of gene expression during receptacle development in cultivars of strawberry.

## Results

### Identification of HKGs with stable expression during receptacle development in strawberry

Among the most frequently used RGs for RT-qPCR in studies of strawberry fruit are the genes encoding *26–18S rRNA*, *Actin*, and *GAPDH*. These genes have been recognized as stably expressed HKGs and historically used as RGs in other plants. Previously, the potential of 16 pre-selected traditional HKGs were evaluated during fruit ripening [14–17]. However, the existence of additional superior RGs among these gene families has not been investigated previously. To address this shortcoming, we identified 6, 6, 13, 3, 16, 19, 8, 42, 102, 54, 3 and 8 members of the *Actin*, *GAPDH*, *Tubulin*, *EF1α*, *SWIB*, *QUL*, *FHA*, *bZip*, *ERF*, *UBC, PDC* and *HISTH4* gene families, respectively, in version 4 of the *F. vesca* genome assembly [25] (Figs. S2, S3, S4, S5, S6, S7, S8, S9, S10, S11, S12 and S13). Here, *26–18S rRNA*, *CHP1*, *ENP1* and *UBQ11* were not analyzed because they were not annotated as a gene in the strawberry genome, or no sequence information is provided in the previous reports. Then, 80 publicly available RNA-seq libraries, which includes data for strawberry receptacle development, were mined. These libraries include four receptacle development experiments for three cultivars of *F. vesca*, comprising ‘Hawaii-4’, ‘Yellow Wonder 5AF7’, and ‘Ruegen’, and five experiments for receptacle development for five cultivars of *F.* × *ananassa*, consisting of ‘Sweet Charlie’, ‘Camarosa’, ‘Toyonoka’, ‘Benihoppe’, and ‘Neinongxiang’. For a detailed description of the RNA-seq samples see Table S1. All 80 libraries were mapped to the *F. vesca* genome assembly v4.0 (Table S1). To identify eligible RGs from the aforementioned HKG families for strawberry receptacle development, we used a similar approach as described by Dekkers et al. [26]. For identification, the expression level and stability of candidate RGs were evaluated: (i) expression abundance, with a cut-off mean FPKM value ≥100, and (ii) expression stability, with a cut-off mean CV value ≤0.2. The thresholds were applied to the mean of the nine experiment data sets. Genes with higher FPKM values showed increased expression abundance and those with a lower CV value were more stably expressed. A total of 15 transcripts from the HKG families showed superior abundance and stability of expression, namely *FveACT6*, *FveTUA2*, *FveEF1ɑ1*, *FveEF1ɑ2*, *FveGPDH4.1*, *FveUBC5*, *FveUBC10*, *FveUBC12*, *FveUBC16*, *FveUBC18*, *FveUBC21*, *FveUBC46*, *FveUBC51*, *FveUBC50* (*FaDBP*) and *FveHISTH4.1* (Fig. S14). Thus, we defined *26-18S rRNA*, *ACT6*, and *GPDH4.1* as the “Commonly used” HKGs set and the remaining eligible genes were defined as the “Traditional” HKGs set. .

### Identification of specific RGs during strawberry receptacle development

**Fig. 1:**
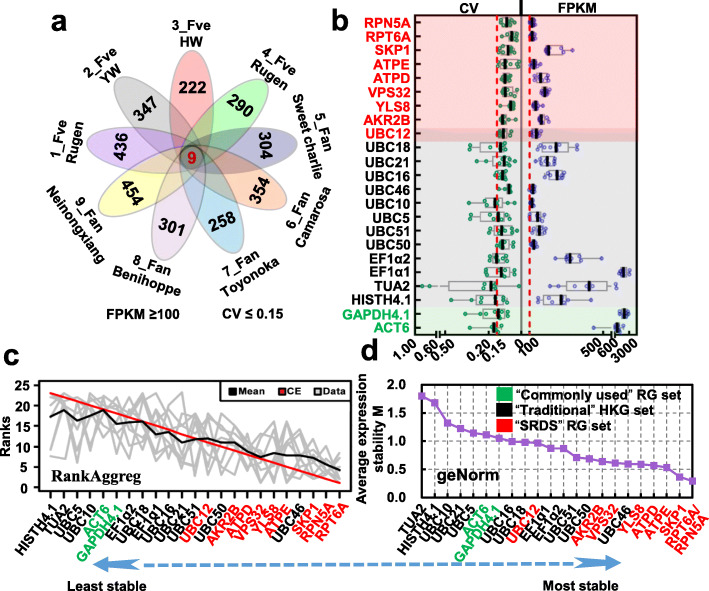
Identification of specific reference genes in strawberry receptacle development based on RNA-seq data. To discover additional superior RGs during receptacle development, we adopted a screening procedure with cut-off values for coefficient of variation (CV) ≤ 0.15 and reads per kilobase per million (FPKM) ≥ 100 in nine RNA-seq data sets that include receptacle development experiments in strawberry. **a** Venn diagram showing nine candidate RGs identified from the complete genome. The numbers represent the gene numbers meet the criteria for the each RNA-seq data set. **b** Statistical analysis of CV and FPKM values of strawberry receptacle development specific (“SRDS”) RGs, “Commonly used” HKGs and “Traditional” HKGs identified from the nine RNA-seq data sets. The CV analysis is shown on the left side of the figure, and the FPKM analysis is shown on the right side of the figure. Each data point in the box-plot is derived from one RNA-seq data set. The horizontal line in the box represents the median. The red dashed lines indicate the cut-off values. **c** Ranking of the candidate RGs into nine lists on the basis of expression stability from CV values in each experiment of the RNA-seq data set. The RankAggreg package for R was used to generate a consensus ranking from the nine lists. The merged list revealed that “SRDS” RGs showed greater expression stability except for *UBC12*. **d** Expression data for the candidate RGs were analyzed using geNorm to evaluate their expression stability by calculating a stability value (M) for each gene. Increase in gene expression stability corresponds with a lower M value. The results were consistent with the ranking of the RGs. The RNA-seq data implied that the expression stability of “SRDS” RGs was superior to that of the “Commonly used” HKGs and “Traditional” HKGs during strawberry receptacle development. The colors indicate different sets of candidate RGs in **b–d**. Note: 26–18S rRNA was not analyzed here because it was not annotated on the *F. vesca* genome assembly v4

To discover additional superior RGs during receptacle development, we adopted stricter screening criteria with cut-off values of CV ≤ 0.15 and FPKM ≥100 for the nine RNA-seq data sets. The thresholds were applied simultaneously to the data sets of the nine experiments. Nine genes were identified from the complete genome by this process (Fig. 1a): Regulatory particle triple-A ATPase protein 6A (*RPT6A*), Regulatory particle non-ATPase protein 5A (*RPN5A*), Vacuolar protein sorting protein 34 (*VPS34*), S-phase-kinase-associated protein 1 (*SKP1*), Ubiquitin-conjugating protein 12 (*UBC12*), ATP synthase subunit δ (*ATPD*), ATP synthase subunit ε (*ATPE*), Ankyrin repeat protein 2B (*AKR2B*), and Yellow-leaf-specific protein 8 (*YLS8*). We designated these genes as “strawberry receptacle development specific (SRDS)” RGs (Table 1, Fig. 1b). Notably, among these nine genes, seven genes are associated with the ubiquitin 26S proteasome system (UPS) (Fig. S15).

**Table 1:** Gene description, primer sequences, amplicon length, and PCR efficiency for candidate RGs and CHS1 selection in strawberry

Gene name	Gene description	Gene ID	Arabidopsis homolog locus	E values	Primer sequence Forward (5′-3′)	Primer sequence Reverse (5′-3′)	Amplicon size (bp)	PCR efficiency (%)	Correlation coefficient ***(R***^***2***^)
RPT6A	26S proteasome regulatory particle AAA-ATPase subunit protein 6A	FvH4_1g03980	AT5G19990	0	GGTTTTGATGGCTACAAATCGT	CCATTCATTTTCTCGGCAATCT	188	100.2	0.998
RPN5A	26S proteasome regulatory particle non-ATPase subunit protein 5A	FvH4_5g27840	AT5G09900	0	GAGGCAATTTAGACGCGCAA	GCTCAAGAATGTCAGTGGCG	107	99.5	0.997
VPS32	Vacuolar protein sorting protein 32	FvH4_1g06720	AT2G19830	6e-65	AAAGCAACGAACATAGATGACG	CTGAACCAATTGGAGTTGACAG	105	100.8	0.998
SKP1	Subunit of SCF complex, S-phase-kinase-associated protein 1	FvH4_1g11300	AT1G75950	9e-67	TCATTTCAGTTCTCTCCACACA	GATCATGTGCTTGATCGTCTG	202	99.9	0.999
AKR2B	Ankyrin repeat protein 2B	FvH4_2g17270	AT2G17390	e-115	CCCAATCCTTTTGATTTCTCGG	TGACTATCAAACTGAGGGACAC	169	101	0.999
YLS8	Yellow leaf specific gene 8	FvH4_3g08480	AT5G08290	2e-80	TTTGCTGTCATTTACCTTGTGG	GTTGATCTTGTTGTTGTTCCCA	146	100.5	0.997
ATPD	ATP synthase subunit δ	FvH4_7g01010	AT5G13450	1e-66	AGTGCCTGCAGATACTAGAAAG	CTTTCACTATTCCCTTATGCGC	176	98.9	0.996
ATPE	ATP synthase subunit ε	FvH4_7g08910	AT5G47030	8e-77	TGAACTCGGCCTCAACTGAC	ACAAGGGAGCACAAAGACCA	114	101.2	0.995
UBC12	Ubiquitin conjugating enzyme E2	FvH4_3g35650	AT5G59300	2e-72	TTATCCTGATGGGCGCGTTT	GTGACTTTCTCACGCAACGG	241	101.7	0.998
UBC5	Ubiquitin conjugating enzyme E2	FvH4_1g16390	AT2G36060	8e-76	ACAAGAGAAGGGTTCGCCAG	AACAAAAGGCGGCAACTGAC	117	99.8	0.994
UBC10	Ubiquitin conjugating enzyme E2	FvH4_2g35960	AT5G25760	9e-87	TGCATTTCAAGACAGGAGAGAT	TAGCCCTACAAACAGACTGAAG	158	99.8	1.000
UBC16	Ubiquitin conjugating enzyme E2	FvH4_5g03910	AT5G53300	1e-83	TTGCTGAAGACATGTTTCACTG	TCAACAGTGAGCAAATCGAAAG	254	98.9	0.993
UBC18	Ubiquitin conjugating enzyme E2	FvH4_7g30920	AT5G53300	5e-83	CCAAAGGTGGCATTTAGAACAA	TCCTTGCTGTTGTCTCATACTT	225	99.8	0.999
UBC21	Ubiquitin conjugating enzyme E2	FvH4_3g40820	AT1G64230	1e-83	GATAGCCCGTATGCAGGTGG	CATCAGGGTTGGGGTCTGTC	225	97.8	0.996
UBC46	Ubiquitin conjugating enzyme E2	FvH4_3g18500	AT1G78870	1e-85	TGCCTCTCGACCCCAAAAAT	GGGAAGGTTACTGTTCGCCA	147	101.8	1.000
UBC50	Ubiquitin conjugating enzyme E2	FvH4_3g25890	AT3G52560	1e-73	GTGGAGAAAAGGGCATCGGA	CGCCCCTCGTGAACAGTATT	120	100	0.997
UBC51	Ubiquitin conjugating enzyme E2	FvH4_6g19850	AT2G36060	8e-76	TCCTTCTCACTTGCCTTCGTC	AGCCTAGCGTCATGGGTACT	121	100.2	0.998
EF1ɑ2	Elongation factor 1-alpha	FvH4_7g20050	AT5G60390	0	GCTTCAAACTCCAAGGATGATC	CTTAACAAAACCAGCATCACCA	233	99.6	0.999
EF1ɑ1	Elongation factor 1-alpha	FvH4_3g33150	AT5G60390	0	ATACAACCCAGACAAAATTGCC	ACCACCGATCTTGTATACATCC	192	101.6	0.995
TUA2	Alpha tubulin like protein	FvH4_1g18660	AT1G50010	0	CTTCAACACCTTCTTCTCCGAG	GATCTCTTTGCCGATGGTGTAG	176	98.8	0.999
HISTH4.1	Histone H4	FvH4_6g14140	AT2G28740	0	TCAAGCGTATCTCCGGTCTC	AGTGTCCTTCCCTGCCTCTT	163	97.8	0.994
UBQ11	Ubiquitin	/			CAGACCAGCAGAGGCTTATCTT	TTCTGGATATTGTAGTCTGCTAGGG	/	98.6	0.995
ENP1	Endoplasmin-like protein	/			GCCACGTCTCTTTGACATTGACT	TTCCGAATGGGCTTTCCA	71	99.3	0.992
CHP1	Conserved hypothetical protein	/			TGCATATATCAAGCAACTTTACACTGA	ATAGCTGAGATGGATCTTCCTGTGA	91	98.9	0.99
ACT6	Actin protein	FvH4_1g23490	AT5G09810	0	GCCAACCGTGAGAAGATG	TCCAGAGTCAAGAACAATACCAG	106	100.8	0.998
26S–18S	18S–26S interspacer ribosomal gene				ACCGTTGATTCGCACAATTGGTCATCG	TACTGCGGGTCGGCAATCGGACG	149	100.6	0.999
GAPDH4.1	Glyceraldehyde-3-phosphate dehydrogenase	FvH4_4g24420	AT1G13440	e-172	TCCATCACTGCCACCCAGAAGACTG	AGCAGGCAGAACCTTTCCGACAG	196	100.8	1.000
CHS1	Chalcone synthase	FvH4_7g01160	AT5G13930	0	ACGCAACAACACACAGCTCC	TTGGGAGGAGTTGCAGTCCC	173	99.1	0.994

Note: “/”: the data is not released in any publicaion

To confirm further the expression stability in strawberry receptacle development, the “SRDS” RGs set were compared with the “Commonly used” and “Traditional” HKGs. We calculated the expression ratio per gene were obtained by dividing the expression value per sample by the average expression level in each experiment set from the RNA-seq data to evaluate expression stability (plotted in Fig. S16). The “Commonly used” HKGs showed considerable variation in expression over the 80 strawberry fruit libraries. In comparison, a majority of “Traditional” HKGs showed greater stability of expression. However, an even higher degree of expression stability was exhibited by the “SRDS” RGs, which suggested that this set contained superior RGs from these candidates (Fig. S16). To test this hypothesis, we ranked the candidate RGs into nine lists according to the expression stability based on the CV value in each experiment set from the RNA-seq data. Discrepancies in the rank positions of candidate RGs were observed among these lists. To provide a consensus, we used RankAggreg, a package for R using a Monte Carlo algorithm and establish a consensus ranking [27], to merge the nine outputs. The merged list revealed that “SRDS” RGs also showed greater expression stability except for *UBC12* (Fig. 1c). Among the “SRDS” RGs, *RPT6A* and *RPN5A* were the top-ranked genes. In contrast, the “Commonly used” HKGs received the lowest rankings, which revealed their inferior expression stability.

The RNA-seq expression data for these candidate RGs were also analyzed using geNorm, which evaluates the expression stability of genes by calculating a stability value (M) for each gene. The greater expression stability of a gene, the lower the M value. A similar ranking trend was obtained in this analysis, although a slight change in the order of RGs in the middle rankings was observed (Fig. 1d). The results of these RNA-seq data analyses implied that on the basis of expression stability the “SRDS” RGs outperformed the “Commonly used” and “Traditional” HKGs in strawberry receptacle development.

### **Detection by RT-qPCR of RGs expression stability in strawberry** receptacle development

**Fig. 2:**
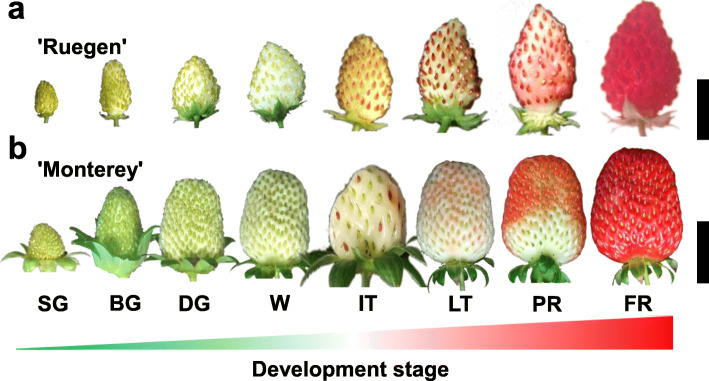
Stages of strawberry fruit development. The receptacle samples were collected at eight visual developmental stages from strawberry ‘Ruegen’ (diploid) (Bar = 1 cm) (**a**) or ‘Monterey’ (octaploid) (Bar = 2 cm) (**b**). SG (small green), BG (big green), DG (degreening), WT (white), IT (initial turning), LT (late turning), PR (partial red), and FR (full red)

To test the hypothesis that the “SRDS” RGs list included superior RGs for strawberry receptacle development, we validated the expression stability of the candidate RGs in strawberry receptacles by RT-qPCR. Eight visual developmental stages for *Fragaria vesca* cultivar ‘Ruegen’ and *F.* × *ananassa* were sampled: small green, big green, degreening, white, initial turning, late turning, partial red, and full red stages (Fig. 2). The quality of the isolated RNA from the fruit samples (Fig. S17) and specificity of RT-qPCR primers (Fig. S18) were thoroughly checked before further processing. For further confirmation, 27 candidate RGs (*26-18S rRNA*, *UBQ11*, *CHP1*, *ENP1* were included in this analysis) were validated by RT-qPCR analysis. For a detailed description of the detection procedure see the Materials and Methods.

**Fig. 3:**
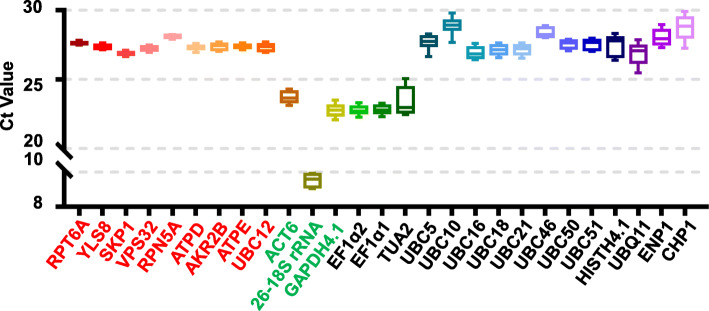
CT analysis of the 23 candidate reference genes in RT-qPCR analysis. The CT values of the 23 candidate RGs were pooled to evaluate their expression profiles. A box-whisker plot showing the CT variation among 16 test samples was generated. The horizontal line in the box represents the median. The upper and lower limits of each box indicate the 25th and 75th percentiles. Whiskers indicate the minimum and maximum values

A flowchart of the procedure to evaluate the expression stability of the candidate RGs in the RT-qPCR analysis was shown in Fig. S19. The cycle threshold (CT) value is an index that represents gene expression in the RT-qPCR analysis. Gene with a lower variation of CT value show more expression stability, and with a high CT value show low expression abundance. If CT value are too high (> 30) or too low (< 15), a gene is generally considered inappropriate as an RG, because it’s unreasonable expression abundance. The CT values for the 23 candidate RGs were pooled to evaluate their expression profiles, and a box-whisker plot showing the CT variation among 16 test samples was generated (Fig. 3). All candidate RGs exhibited appropriate CT values except *26–18S rRNA*. The average CT values ranged from 9.51 (*26–18S rRNA*) to 28.91 (*UBC10*). The “SRDS” RGs showed appropriate average CT values ranging from 26.64 to 27.88, and lower expression variation (less than 0.76 cycles) compared with “Traditional” and “Commonly used” HKGs [expression variation ranged from 0.86 cycles (*UBC50*) to 2.58 cycles (*TUA2*)] (Fig. 3). These results indicated that the “SRDS” RGs showed greater expression stability than “Traditional” and “Commonly used” HKGs and were more suitable for normalization of genes with low- to medium-abundance expression profiles.

**Fig. 4:**
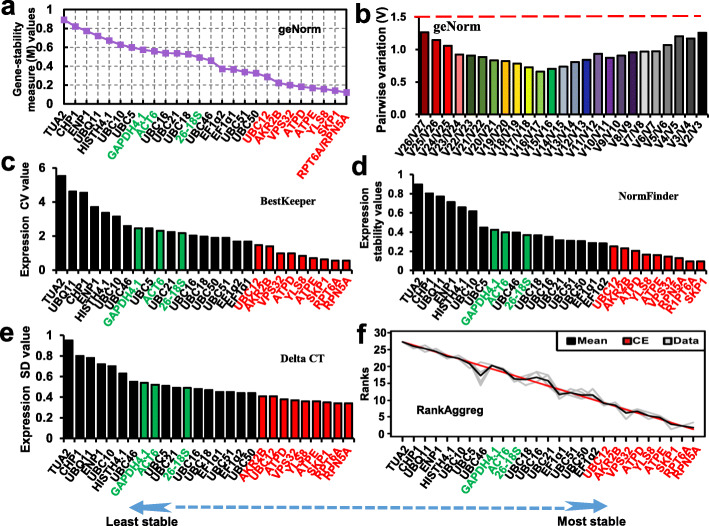
Expression stability of candidate reference genes of ‘Ruegen’ and ‘Monterey’ in combination analyzed by RT-qPCR. To evaluate the expression stability of the RGs, gene-stability measure (M), stability, coefficient of variation (CV), and standard deviation (SD) values were calculated using geNorm (**a**), BestKeeper (**c**), NormFinder (**d**) and Delta CT (**e**). A lower value indicates greater stability of expression. The RankAggreg package for R was employed to merge the stability measurements obtained from the four tools using a Monte Carlo algorithm and to establish a consensus ranking of the RGs (**f**). The pairwise variation (*V*_*n*_/*V*_*n* + 1_) was calculated to determine the optimal number of RGs for normalization of gene expression (**b**)

In addition, we evaluated and ranked the candidate RG expression stability in all samples, considering ‘Ruegen’ and ‘Monterey’ together, on the basis of different stability indices calculated using four software programs (geNorm, NormFinder, BestKeeper, and Delta CT), which have been widely applied in studies of internal reference evaluation. The results were consistent in revealing that “SRDS” RGs showed superior expression stability compared with that of “Traditional” and “Commonly used” HKGs (Fig. 4a, c, d, e). Among these genes, *RPT6A* and *RPN5A* were the most stable RGs. Strikingly, the ranking of “Commonly used” HKGs in the lowest ranks revealed their inferior expression stability compared with “SRDS” RGs. We next used RankAggreg to merge the four rankings (Fig. 4f). The results corroborated the aforementioned rankings from geNorm, NormFinder, BestKeeper, and Delta CT analysis (Fig. 4), and also corresponded with the results of the RNA-seq data analysis (Fig. 1).

Normalization of gene expression using multiple RGs may increase measurement accuracy in RT-qPCR analyses. Thus, we investigated the optimal number of RGs for normalization in strawberry receptacle development. This analysis was performed by computing the pairwise variation (PV; *V*_*n*_/*V*_*n* + 1_) using geNorm software. Once the PV value for *n* genes is below a cutoff of 0.15, which is a recommended threshold that is universally accepted, additional genes are considered not to improve normalization. The pairwise variation *V*2/3 value (0.126) was less than the threshold (Fig. 4b). Therefore, two RGs (*RPT6A* and *RPN5A*) in combination were sufficient for normalization in strawberry receptacle development. We next analyzed the stability and optimal number of candidate RGs in strawberry receptacle development, in separate analyses for ‘Ruegen’ and ‘Monterey’, which showed similar results for each cultivar (Figs. S20 and S21). These results indicated that the recommended “SRDS” RGs were applicable to different cultivars of strawberry.

### Validation of recommended RGs for normalization of *FveCHS1* expression

**Fig. 5:**
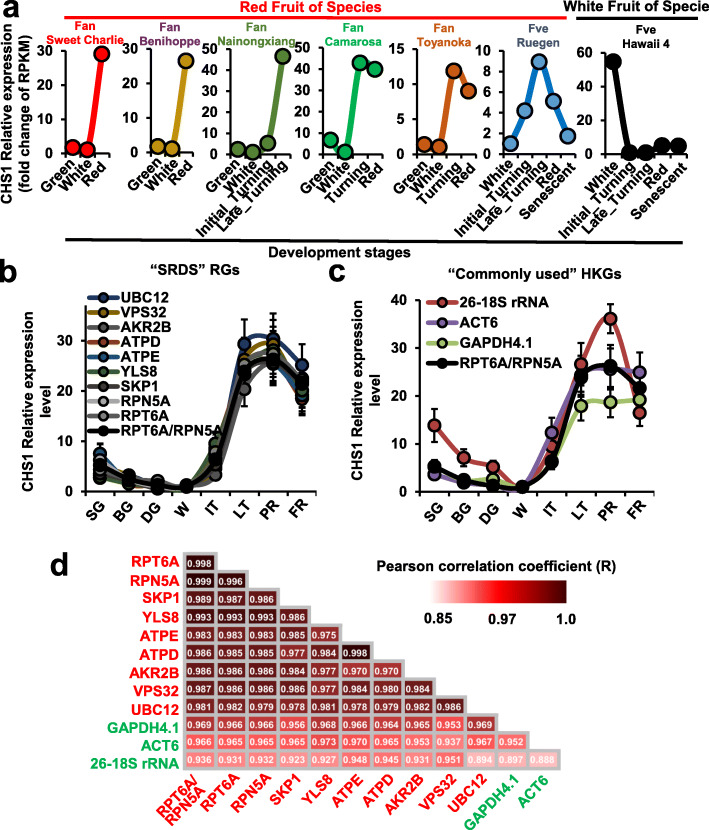
Validation of recommended reference genes by normalization of *FveCHS1* expression in ‘Ruegen’. **a** *FveCHS1* expression was strongly associated with anthocyanin content in cultivars of strawberry with red fruit or white fruit based on RNA-seq analysis. *FveCHS1* expression was analyzed to validate the reliability of the recommended RGs by RT-qPCR (**b**, **c**). **b** Relative expression levels of *FveCHS1* derived by normalization against an individual “SRDS” RG or combination of *RPT6A* and *RPN5A* indicated similar trends from RNA-seq data during receptacle development. **c** Relative expression level of *FveCHS1* when normalized against “Commonly used” HKGs. The results confirmed that an individual “SRDS” RG or combination of *RPT6A* and *RPN5A* were superior to “Commonly used” HKGs for normalization of gene expression during receptacle development of strawberry. The receptacle samples were collected at eight visual developmental stages from strawberry ‘Ruegen’ in **c** and **d**. SG (small green), BG (big green), DG (degreening), WT (white), IT (initial turning), LT (late turning), PR (partial red), and FR (full red). **d** Pairwise correlation analyses were performed by calculating the Pearson correlation coefficient (R) to estimate the similarity of the trend in *CHS1* relative expression level when normalized against candidate RGs

Chalcone synthase (CHS) is involved in anthocyanin biosynthesis during fruit ripening. Suppression of CHS activity in strawberry fruit by transient or stable transgenesis results in a substantial decrease in anthocyanin content [28]. By conjoint analysis of the *F. vesca* genome and RNA-seq data, we detected expression of a *CHS* gene (*FveCHS1*) that was strongly associated with anthocyanin content in cultivars of strawberry with red fruit. In detail, the expression of *FveCHS1* in the receptacle slightly decreased after the green stage, increased sharply and peaked at the turning stage, and thereafter slightly declined at the red stage (Fig. 5a). By contrast, this expression profile was absent in cultivars with white fruit, such as ‘Hawaii-4’ (Fig. 5a). These results suggested that *FveCHS1* facilitated anthocyanin biosynthesis during receptacle ripening in cultivars of strawberry. Thus, the *FveCHS1* expression profile was employed to validate the reliability of the recommended RGs by RT-qPCR. Unsurprisingly, relative expression levels of *FveCHS1* obtained by normalization against combination of *RPT6A* and *RPN5A*, the preferred recommendation above, indicated similar trends to those from the RNA-seq data analysis during receptacle development in cultivars of strawberry with red fruit (Fig. 5b). Meanwhile, the similar results also were exhibited by normalization against individual “SRDS” RGs (Fig. 5b). However, the trend in *FveCHS1* relative expression level during receptacle development differed to varying degrees when normalized against “Commonly used” HKGs (Fig. 5c). For instance, when *ACT6* was used for normalization, the *FveCHS1* relative expression level at the FR stage was equal to that at the LT stage; a similar alteration was observed when *GAPDH4.1* was used for normalization; although the overall trend was consistent, the extent of variation in *FveCHS1* relative expression level was notably increased when *26–18S rRNA* was used for normalization (Fig. 5c). Pairwise correlation analyses were performed by calculating the Pearson correlation coefficient (R) to estimate the similarity of the trend in *CHS1* relative expression level when normalized against candidate RGs. Genes with higher R values showed the more closely resemble normalization effect. The result revealed that a considerably high survival R values were obtained when the *CHS1* relative expression level was normalized by individual “SRDS” RGs compared to normalization by combination of *RPT6A* and *RPN5*, which as a reference standard*.* However, the R values were relatively low when in the *CHS1* relative expression level was normalized against “Commonly used” HKGs compared to normalization by combination of *RPT6A* and *RPN5* (Fig. 5d). Thus, in practical application, these results confirmed that use combination of *RPT6A* and *RPN5A* or individual “SRDS” RGs were superior to use of “Commonly used” HKGs for normalization of gene expression during receptacle development in strawberry cultivars.

## Discussion

The inaccurate normalization are inadequate for analyzing gene expression simultaneously track more transcripts with greater sensitivity. In a species with diverse cultivars like strawberry, identifying a universally applicable constitutive RG transcripts in fruit development study increasing the challenge. To date, three traditional HKGs that encode the 26–18S rRNA, ACTIN, and GAPDH are most frequently used for RT-qPCR in strawberry fruit studies. Unfortunately, these RGs are utilized generally without validation of their stability, while directly learned from experience in other plants. Constitutive expression of some HKG transcripts, including the three “Commonly used” HKGs in strawberry, are often been queried, because they are experimentally validated as inappropriate RGs. For instance, traditional HKGs from a developmental series of *Arabidopsis* seed and pollen samples show highly variable expression [9]. Specifically, the ACTIN transcript as a research case has been widely tested and shown to be inconsistent in poplar [29], potato [30], and rice [31]. It improves the idea of setting aside the preconception that HKGs are evenly expressed under difference species, tissues and certain conditions is generally accepted. To address this shortcoming, several researches have taken the tries that selected several members of HKGs were assessed during strawberry fruit ripening, of which *RIB413* (*26–18S rRNA*), *ACTIN*, *HISTH4*, *DBP* (*UBC50*) and *UBQ11* were recommended as appropriate RGs [14–17]. However, in this study showed that these recommended transcripts are not stably expressed and are inappropriate for normalization during fruit development in strawberry cultivars (Figs. 1, 4 and 5). Similar findings were also observed in other Rosacea species like peach [32]. The results from these rosaceous crops revealed that few transcripts are truly stable expression over broad ranges of fruit developmental states. Although, the same effort was worked in this study, we selected a total of the addition 13 “Traditional” HKGs transcripts from the HKG families showed more stability of expression, the weaknesses for this method is restriction in the scope of selecting the candidates evaluated.

The promising mRNAs for normalization proved to be those identified from transcriptomic sequencing efforts, and represent transcripts from some uncharacterized genes [33]. Such genome-based approaches have proven useful in *Arabidopsis* [9] where several unexploited transcripts were identified that improved the precision of RT-qPCR analysis over traditional reference transcripts. Previously, Clancy et al. (2013) have been attempted to screen the superior RGs during strawberry fruit ripening by merging digital gene expression data with expression profiling, and *CHP1* and *ENP1* were considered as constitutively expressed RGs [20]. However, these potential constitutive candidates needed to be confirmed through more transcriptome sets, as a small sample size could include bias. In this study, extensive RNA-seq data (80 libraries) previously generated for receptacle development in strawberry were mined to validate superior RGs. These RNA-seq data including nine experiments set with eight cultivars of strawberry were particularly noteworthy since it rendered our more possibly to obtain superior RGs which are universally applied during fruit development in diverse strawberry cultivars (Table S1). Nine “SRDS” RGs were identified from the complete genome by a very strict screening criteria (Fig. 1). Specifically, the use of multiple contrasting detection methods is strength of this work. Viewed as groups, ranking the candidates with four RNA-seq analysis (CV, FPKM, geNorm and expression ratio assessment) and five RT-qPCR statistical methods (CT pooled, geNorm, BestKeeper, NormFinder and Delta assessment) is largely consistent. Finally, the reliability of the recommended RGs were once again validated by normalization of *CHS1* expression during strawberry receptacle development. This study merges these three independent assessment strategy sets were consistently revealed that the “SRDS” transcripts as optimal RGs showed most stability expression and improved the precision of RT-qPCR, compared with that the “Commonly used” and “Traditional” HKGs, including *CHP1* and *ENP1*, are shown to be less reliable. It forcefully demonstrates that these results are reliable and repeatable. Although it also exist at slightly different among the ranked candidates in orders due to various algorithms of methods were used for assessment [10–13].

Notably, these novel transcripts were recommended in this study are never expanded as RGs in any plant. Surprisingly, the majority of stably expressed candidates were associated with the ubiquitin proteasome system (UPS), which is highly conserved in eukaryotes and is important for plant development. Among these, seven of the nine “SRDS” RGs were associated with the UPS proteolytic pathway (Fig. S15), which revealed that this pathway continuously operates as a basic cellular function throughout strawberry receptacle development. These candidate RGs covered most of the processes of the UPS, including proteasome assembly, ubiquitination, energy supply, and ubiquitin recycling (Fig. S15). *UBC* genes encode E2 ubiquitin-conjugating enzymes, which catalyze the transfer of activated ubiquitin from E1 to the active site cysteine of the E2 via a trans (thio) esterification reaction. This reaction is achieved by connecting the SKP1-Cullin-F-box (SCF) complex to approximate the substrate. SKP is a core component of the SCF complex and serves as an adaptor protein to connect the F-box and Cullin protein to recognize the target protein during ubiquitination [34]. Once a protein is tagged with a single ubiquitin, the signal is activated to attach additional ubiquitin molecules. This results in a polyubiquitin chain, which is bound to the 26S proteasome, allowing the latter to degrade the tagged protein. *RPN* and *RPT* genes encode subunits for assembly of the 26S proteasome [35]. The content of ubiquitin protein is dynamically balanced in plant cells. It requires the deubiquitination of the Endosomal Sorting Complex Required for Transports (ESCRTs) complex to recycle ubiquitin and maintain cytoplasmic levels of free ubiquitin [36]. VPS34 is a subunit of ESCRT-III, which is a complex for constituting the ESCRTs. ESCRTs profoundly shape signal transduction is based on the degradative sorting of ubiquitinated membrane proteins and deubiquitylation [37]. The UPS requires abundant ATP for normal operation [38]. F_0_F_1_-ATP synthase, a large multi-subunit complex within the inner mitochondrial (mt) membrane, is the site of conversion of the energy stored in complex molecules into ATP. In the present study, two fruit-specific RGs, *ATPD* and *ATPE*, encode two different subunits of the mt ATP synthase, namely the δ (ATP5) and the ε (ATP3) subunits. The role of subunit δ is to combine the pore-forming *c* and *a* subunits in the last step of ATP synthase assembly. Subunit ε combines with subunit γ to form a central stalk that non-covalently interacts with the membrane-embedded, hydrophobic *c*-ring [39, 40]. In addition, two uncorrelated UPS genes, *AKR2B* and *YLS8*, were also identified as receptacle-specific RGs in the current study. Membrane proteins are usually synthesized on free ribosomes in the cytoplasm, subsequently received by molecular chaperones before being targeted to their membranes. In *Arabidopsis*, *AKR2B* and its close homolog, *AKR2A*, are cytosolic factors that recognize the protein-targeting signals and deliver them to the chloroplast outer membrane [41]. *YLS8* encodes a mitosis protein, but its precise function is unknown. *YLS8* was previously observed to outperform traditional RGs, with regard to expression stability, throughout development in *Arabidopsis* [9]. Subsequently, in several plant species, *YLS8* has been assessed and confirmed to show enhanced expression stability during development compared with conventional RGs using transcription data [42, 43].

In general, the nine “SRDS” RGs identified in the present study perform basic physiological functions in plant cells and participate throughout the entire plant development period, which thus provides a biological basis for their stable expression.

## Conclusions

In this study, we identified nine RGs specific to receptacle development in strawberry based on genome-wide analysis. The results of the multifaceted assessment consistently showed that the novel RGs showed improved expression stability compared with that of the “Commonly used” and “Traditional” HKGs in transcriptome and RT-qPCR analyses. Notably, the majority of stably expressed genes were associated with the UPS proteolytic pathway. Among these, two 26S proteasome subunits, *RPT6A* and *RPN5A,* are recommended as the optimal RGs combination. This finding provides additional useful and reliable RGs as resources for the accurate study of gene expression during receptacle development in cultivars of strawberry.

## Methods

### Plant material

Plants of *Fragaria vesca* cultivar ‘Ruegen’ and *F.* × *ananassa* cultivar ‘Monterey’ from the germplasm garden of College of Horticulture, Fujian Agriculture and Forestry University (Fuzhou City, Fujian Province, China) were prepared for this study. Plants were cultured in a greenhouse in Fuzhou, China. Growing conditions were maintained at 25 °C under a 16 h photoperiod providing 120 μmol m^− 2^ s^− 1^ irradiance. We modified a previously reported six-stage division of strawberry fruit development [44] and exploited higher-density sampling. Fruit were harvested at eight developmental stages: small green (7 days after flowering, DAF), big green (14 DAF), degreening (18 DAF), white (21 DAF), initial turning (23 DAF), late turning (25 DAF), partial red (27 DAF), and full red (29 DAF) stages (Fig. 2). The achenes were separated from the receptacle. Each sample were randomly selected from eight fruits, then receptacle was cut into small pieces for mixing, immediately frozen in liquid nitrogen, and stored at − 80 °C before isolating the total RNA. For all samples, four biological replicates were performed.

### Identification and phylogenetic analysis of the HKG families in strawberry

Multiple strategies were used to search for members of the HKG families in strawberry. (1) Keyword searches were performed against the annotated strawberry protein databases; (2) hidden Markov model (HMM) searches with the conservative domain HMM profile for proteins, and all significant hits (HMMER E-value < e^− 5^) were subsequently evaluated; (3) *Arabidopsis thaliana* HKG sequences were used as queries in exhaustive BLASTP searches with standard parameters of the strawberry protein databases. All candidate protein sequences were analyzed using the InterProScan5 database (http://www.ebi.ac.uk/interpro/search/sequence-search) to verify the presence of the necessary domain; protein sequences lacking this domain were removed (Figs. S2, S3, S4, S5, S6, S7, S8, S9, S10, S11, S12 and S13). The amino acid sequences of the selected HKGs were aligned using ClustalX [45]. Phylogenetic trees were constructed via the maximum likelihood method in MEGAX using the best amino acid substitution model [46]. Bootstrap analysis was performed with 1000 iterations using the parameters *p*-distance and partial deletion with 50% site coverage cutoff option. Strawberry HKGs were named based on the nomenclature established for *Arabidopsis* (Figs. S2, S3, S4, S5, S6, S7, S8, S9, S10, S11, S12 and S13).

### Transcript assembly and quantification for RNA-seq

Eighty available RNA-seq libraries, which includes studies of strawberry receptacle development, from the National Center of Biotechnology Information database (NCBI) (https://www.ncbi.nlm.nih.gov/) were mined. All libraries data were mapped to the *F. vesca* reference genome assembly v4.0 using the HISAT2 aligner [47]. The percentage of uniquely aligned reads varied among samples ≥77.55% (Table S1), which indicated that the RNA-seq data quality was acceptable. The StringTie software was used to calculate the fragment per kilobase of transcript per million mapped reads (FPKM) values [47]. Genes with a high FPKM value showed a high abundance of transcripts.

### RNA extraction and cDNA preparation

Total RNA was extracted using the TRIzol reagent (TaKaRa, Dalian, China) in accordance with the manufacturer’s instructions. RNA samples were assessed with ratios OD 260/280 > 2.0 and OD 260/230 > 1.8 (Fig. S17). To eliminate genomic DNA contamination, equal amounts of total RNA (2 μg) in all RNA samples were treated with DNAase I (Fig. S17). Then, cDNA was synthesized using the PrimeScript™ RT Reagent Kit (Perfect Real Time; TaKaRa, Dalian, China). RNA extraction and cDNA synthesis from all samples was performed with four biological replicates.

### Primer design, amplification specificity, and efficiency analysis for candidate RGs in RT-qPCR

We used the primers for *GAPDH4.1* [6, 7], *ACT6* [4, 5], and *26–18S rRNA* [2, 3] confirmed in previous studies and widely used in studies of strawberry fruit, and designed new primers for the remaining candidate RGs using Premier 5.0 software (Table 1). The PCR efficiency and specificity of the primers were thoroughly evaluated (Table 1, Fig. S18). In all indices the primers met the MIQE guidelines [48], which provide guidelines on the minimum information for publication of RT-qPCR experiments. Lin-RegPCR software was used to evaluate efficiency by calculating correlation coefficients (*R*^2^) and amplification efficiency of the candidate RG primers in RT-qPCR (Table 1) [49]. The specificity of the primers for the candidate RGs was verified as follows. The presence of a single peak in the melting curve was confirmed after RT-qPCR amplification (Fig. S18a). Subsequently, the amplified products were analyzed by 2.5% agarose gel electrophoresis, and only one band of the expected product size was detected (Fig. S18b).

To assay for gene expression, 20 μl of reaction mixture corresponded to 5 ng total cDNA in one PCR reaction system (TaKaRa SYBR® PrimeScript™ RT-PCR Kit (Perfect Real Time)). The reaction was carried out using a Roche LightCycler® 480II real-time PCR cycler (Roche Diagnostics, Mannheim, Germany) in accordance with the kit manufacturer’s instructions (TaKaRa, Dalian, China). Reaction mixtures were incubated for 10 min at 95 °C for pre-incubation followed by 45 amplification cycles of 15 s at 95 °C, 15 s at 60 °C, and 20 s at 72 °C. A dissociation curve was generated from 60 to 95 °C. Four biological replicates were amplified for all samples. Expression levels of candidate RGs in all samples were determined on the basis of their quantification cycle values (CT). The ∆∆C_q_ method was used to determine *CHS1* mRNA levels among samples.

### Statistical analysis of the expression stability of the candidate RGs

To determine the expression stability of the candidate RGs, gene-stability measure (M), stability (Stab), coefficient of variance (CV), and standard deviations (SD) values were calculated using geNorm [10], BestKeeper [11], NormFinder [12], and Delta CT [13] software, respectively. Increasing stability of gene expression corresponds to lower M, Stab, CV, and SD values. The RankAggreg package for R was employed to merge the stability measurements obtained from the four tools using a Monte Carlo algorithm and establish a consensus ranking of RGs [27]. The rankings from the four terms M, Stab, CV, and SD were used as input.

## Supplementary Information

**Additional file 1: Figure S1.** The structure of strawberry fruit in ‘Ruegen’. **Figure S2.** Identification, phylogenetic and domain analyses of the *Actin* gene family in strawberry and *Arabidopsis*. **Figure S3.** Identification, phylogenetic and domain analyses of the *GAPDH* gene family in strawberry and *Arabidopsis*. **Figure S4.** Identification, phylogenetic and domain analyses of the *Tubulin* gene family in strawberry and *Arabidopsis*. **Figure S5.** Identification, phylogenetic and domain analyses of the *EF1α* gene family in strawberry and *Arabidopsis*. **Figure S6.** Identification, phylogenetic and domain analyses of the *QUL* gene family in strawberry and *Arabidopsis*. **Figure S7.** Identification, phylogenetic and domain analyses of the *SWIB* gene family in strawberry and *Arabidopsis*. **Figure S8.** Identification, phylogenetic and domain analyses of the *FHA* gene family in strawberry and *Arabidopsis*. **Figure S9.** Identification, phylogenetic and domain analyses of the *UBC* gene family in strawberry and *Arabidopsis*. **Figure S10.** Identification, phylogenetic and domain analyses of the *AP2/ERF* gene family in strawberry and *Arabidopsis*. **Figure S11.** Identification, and phylogenetic and domain analyses of the *bZip* gene family in strawberry and *Arabidopsis*. **Figure S12.** Identification, phylogenetic and domain analyses of the *PDC* gene family in strawberry and *Arabidopsis*. **Figure S13.** Identification, phylogenetic and domain analyses of the *HISTH4* gene family in strawberry and *Arabidopsis*. **Figure S14.** Identification of qualified HKGs in strawberry receptacle development based on RNA-seq data. **Figure S15.** Schematic illustration of the cellular functions of nine “SRDS” RGs. **Figure S16.** Relative expression of candidate reference genes from RNA-seq data. **Figure S17.** Strawberry receptacle RNA sample quality assessment. **Figure S18.** Specificity assessment of RT-qPCR primers. **Figure S19.** Flow chart showing procedure for RT-qPCR analysis of candidate reference genes during strawberry receptacle development. **Figure S20.** Expression stability of candidate reference genes of ‘Ruegen’ analyzed by RT-qPCR. **Figure S21.** Expression stability of candidate reference genes of ‘Monterey’ analyzed by RT-qPCR. **Table S1.** Description of 80 samples with receptacle development for RNA-seq in strawberry. **Table S2.** The information of HKG families in strawberry and *Arabidopsis*.

## Data Availability

Genomic and publicly available annotated protein databases for strawberry (*F. vesca*) were downloaded from the Genome Database for Rosaceae (GDR; https://www.rosaceae.org/). The data for *Arabidopsis* were downloaded from the *Arabidopsis* Information Resource (https://www.arabidopsis.org/). The gene identity information is listed in the supplemental material (Table S2).

## Abbreviations

RT-qPCR: Reverse transcription–quantitative real time PCR
RG: Reference gene
HKG: Traditional kousekeeping gene
GAPDH: Glyceraldehyde-3-phosphate dehydrogenase
RNA-seq: RNA sequencing
DAF: Days after flowering
FPKM: Fragment per kilobase of transcript per million mapped reads
R2: Correlation coefficients
CT: Quantification cycle values
CV: Coefficient of variance
SD: standard deviations
RPT6A: Regulatory particle triple-A ATPase protein 6A
RPN5A: Regulatory particle non-ATPase protein 5A
VPS34: Vacuolar protein sorting protein 34
SKP1: S-phase-kinase-associated protein 1
UBC12: Ubiquitin-conjugating protein 12
ATPD: ATP synthase subunit δ
ATPE: ATP synthase subunit ε
AKR2B: Ankyrin repeat protein 2B
YLS8: Yellow-leaf-specific protein 8

## References

[1] Shulaev V, Sargent DJ, Crowhurst RN, Mockler TC, Folkerts O, Delcher AL (2011). The genome of woodland strawberry (*Fragaria vesca*). Nat Genet.

[2] Medinapuche L, Molinahidalgo FJ, Boersma M, Schuurink RC, Lópezvidriero I, Solano R (2015). An R2R3-MYB transcription factor regulates eugenol production in ripe strawberry fruit receptacles. Plant Physiol.

[3] Medinapuche L (2014). MYB10 plays a major role in the regulation of flavonoid/phenylpropanoid metabolism during ripening of *Fragaria × ananassa* fruits. J Exp Bot.

[4] Koehler G, Wilson RC, Goodpaster JV, Sønsteby A, Lai X, Witzmann FA (2012). Proteomic study of low-temperature responses in strawberry cultivars (*Fragaria x ananassa*) that differ in cold tolerance. Plant Physiol.

[5] Li D, Mou W, Xia R, Li L, Zawora C, Ying T (2019). Integrated analysis of high-throughput sequencing data shows abscisic acid-responsive genes and miRNAs in strawberry receptacle fruit ripening. Horticulture Res.

[6] Vallarino JG, Osorio S, Bombarely A, Casañal A, Cruzrus E, Sánchezsevilla JF (2015). Central role of FaGAMYB in the transition of the strawberry receptacle from development to ripening. New Phytol.

[7] Xu W, Peng H, Yang T, Whitaker B, Huang L, Sun J (2014). Effect of calcium on strawberry fruit flavonoid pathway gene expression and anthocyanin accumulation. Plant Physiol Biochem.

[8] Huggett J, Dheda K, Bustin S, Zumla A (2005). Real-time RT-PCR normalisation; strategies and considerations. Genes Immun.

[9] Czechowski T, Stitt M, Altmann T, Udvardi MK, Scheible W-R (2005). Genome-wide identification and testing of superior reference genes for transcript normalization in *Arabidopsis*. Plant Physiol.

[10] Vandesompele J, De Preter K, Pattyn F, Poppe B, Van Roy N, De Paepe A (2002). Accurate normalization of real-time quantitative RT-PCR data by geometric averaging of multiple internal control genes. Genome Biol.

[11] Pfaffl MW, Tichopad A, Prgomet C, Neuvians TP (2004). Determination of stable housekeeping genes, differentially regulated target genes and sample integrity: BestKeeper–excel-based tool using pair-wise correlations. Biotechnol Lett.

[12] Andersen CL, Jensen JL, Ørntoft TF (2004). Normalization of real-time quantitative reverse transcription-PCR data: a model-based variance estimation approach to identify genes suited for normalization, applied to bladder and colon cancer data sets. Cancer Res.

[13] Hellemans J, Mortier G, De Paepe A, Speleman F, Vandesompele J (2007). qBase relative quantification framework and software for management and automated analysis of real-time quantitative PCR data. Genome Biol.

[14] Amil-Ruiz F, Garrido-Gala J, Blanco-Portales R, Folta KM, Muñoz-Blanco J, Caballero JL (2013). Identification and validation of reference genes for transcript normalization in strawberry (*Fragaria× ananassa*) defense responses. PLoS One.

[15] Galli V, Borowski JM, Perin EC, da Silva MR, Labonde J, dos Santos PI (2015). Validation of reference genes for accurate normalization of gene expression for real time-quantitative PCR in strawberry fruits using different cultivars and osmotic stresses. Gene.

[16] Yunting Z, Xiaorui P, Yi L, Yali L, Ya L, Xiaorong W (2018). Evaluation of suitable reference genes for qRT-PCR normalization in strawberry ( Fragaria × ananassa ) under different experimental conditions. BMC Mol Biol.

[17] Schaart JG (2004). Towards consumer-friendly cisgenic strawberries which are less susceptible to Botrytis cinerea.

[18] Narsai R, Ivanova A, Ng S, Whelan J (2010). Defining reference genes in *Oryza sativa* using organ, development, biotic and abiotic transcriptome datasets. BMC Plant Biol.

[19] Libault M, Thibivilliers S, Bilgin DD, Radwan O, Benitez M, Clough SJ (2008). Identification of four soybean reference genes for gene expression normalization. Plant Genome J.

[20] Clancy MA, Rosli HG, Chamala S, Barbazuk WB, Civello PM, Folta KM (2013). Validation of reference transcripts in strawberry (Fragaria spp.). Mol Gen Genomics.

[21] Sánchez-Sevilla JF, Vallarino JG, Osorio S, Bombarely A, Posé D, Merchante C (2017). Gene expression atlas of fruit ripening and transcriptome assembly from RNA-seq data in octoploid strawberry (*Fragaria×ananassa*). Sci Rep.

[22] Hawkins C, Caruana J, Li J, Zawora C, Darwish O, Wu J (2017). An eFP browser for visualizing strawberry fruit and flower transcriptomes. Horticulture Res.

[23] Hu P, Gang L, Xia Z, Zhao F, Li L, Zhou H (2018). Transcriptome profiling by RNA-Seq reveals differentially expressed genes related to fruit development and ripening characteristics in strawberries (*Fragaria×ananassa*). Peerj.

[24] Wang QH, Zhao C, Zhang M, Li YZ, Shen YY, Guo JX (2017). Transcriptome analysis around the onset of strawberry fruit ripening uncovers an important role of oxidative phosphorylation in ripening. Sci Rep.

[25] Edger PP, Vanburen R, Colle M, Poorten TJ, Wai CM, Niederhuth CE (2018). Single-molecule sequencing and optical mapping yields an improved genome of woodland strawberry (*Fragaria vesca*) with chromosome-scale contiguity. Gigascience.

[26] Dekkers BJ, Willems L, Bassel GW, van Bolderen-Veldkamp RM, Ligterink W, Hilhorst HW (2012). Identification of reference genes for RT–qPCR expression analysis in *Arabidopsis* and tomato seeds. Plant Cell Physiol.

[27] Pihur V, Datta S, Datta S (2009). RankAggreg, an R package for weighted rank aggregation. BMC Bioinform.

[28] Lunkenbein S, Coiner H, de Vos CR, Schaart JG, Boone MJ, Krens FA (2006). Molecular characterization of a stable antisense chalcone synthase phenotype in strawberry (*Fragaria × ananassa*). J Agric Food Chem.

[29] Brunner AM, Yakovlev IA, Strauss SH (2004). Validating internal controls for quantitative plant gene expression studies. BMC Plant Biol.

[30] Nicot N, Hausman J-F, Hoffmann L, Evers D (2005). Housekeeping gene selection for real-time RT-PCR normalization in potato during biotic and abiotic stress. J Exp Bot.

[31] Jain M, Nijhawan A, Tyagi AK, Khurana JP (2006). Validation of housekeeping genes as internal control for studying gene expression in rice by quantitative real-time PCR. Biochem Biophys Res Commun.

[32] Tong Z, Gao Z, Wang F, Zhou J, Zhang Z (2009). Selection of reliable reference genes for gene expression studies in peach using real-time PCR. BMC Mol Biol.

[33] Folta KM, Clancy MA, Chamala S, Brunings AM, Dhingra A, Gomide L (2010). A transcript accounting from diverse tissues of a cultivated strawberry. Plant Genome.

[34] Silvia J, Zamira A, Concepción M, Gema LT, Pacios LF, Pozo JC (2010). Del: the *Arabidopsis* cell cycle F-box protein SKP2A binds to auxin. Plant Cell.

[35] Förster F, Lasker K, Beck F, Nickell S, Sali A, Baumeister W (2009). An atomic model AAA-ATPase/20S core particle sub-complex of the 26S proteasome. Biochem Biophys Res Commun.

[36] Qing-Tao S, Schuh AL, Yuqing Z, Kyle Q, Lei W, Michael H (2014). Structural analysis and modeling reveals new mechanisms governing ESCRT-III spiral filament assembly. J Cell Biol.

[37] Katsiarimpa A, Anzenberger F, Schlager N, Neubert S, Hauser M-T, Schwechheimer C (2011). The *Arabidopsis* deubiquitinating enzyme AMSH3 interacts with ESCRT-III subunits and regulates their localization. Plant Cell.

[38] Dametto A, Buffon G (2015). Blasi ÃdADR, Sperotto RA: Ubiquitination pathway as a target to develop abiotic stress tolerance in rice. Plant Signal Behav.

[39] Rühle T, Leister D (2015). Assembly of F 1 F 0 -ATP synthases. BBA-Bioenergetics.

[40] Robison MM, Xingyuan L, Smid MPL, Adel Z, Wolyn DJ (2009). Antisense expression of mitochondrial ATP synthase subunits OSCP (ATP5) and gamma (ATP3) alters leaf morphology, metabolism and gene expression in *Arabidopsis*. Plant Cell Physiol.

[41] Kim DH, Park MJ, Gwon GH, Silkov A, Xu ZY, Yang EC (2014). An Ankyrin repeat domain of AKR2 drives chloroplast targeting through coincident binding of two chloroplast lipids. Dev Cell.

[42] Ma R, Xu S, Zhao Y, Xia B, Wang R (2016). Selection and validation of appropriate reference genes for quantitative real-time PCR analysis of gene expression in *Lycoris aurea*. Front Plant Sci.

[43] Migocka M, Papierniak A (2011). Identification of suitable reference genes for studying gene expression in cucumber plants subjected to abiotic stress and growth regulators. Mol Breed.

[44] Fait A, Hanhineva K, Beleggia R, Dai N, Rogachev I, Nikiforova VJ (2008). Reconfiguration of the achene and receptacle metabolic networks during strawberry fruit development. Plant Physiol.

[45] Thompson JD, Gibson TJ, Higgins DG. Multiple sequence alignment using ClustalW and ClustalX. Curr Protoc Bioinformatics. 2002; Chapter 2(Unit 2):Unit 2.3. 10.1002/0471250953.bi0203s00 18792934

[46] Kumar S, Stecher G, Li M, Knyaz C, Tamura K (2018). MEGA X: molecular evolutionary genetics analysis across computing platforms. Mol Biol Evol.

[47] Pertea M, Kim D, Pertea GM, Leek JT, Salzberg SL (2016). Transcript-level expression analysis of RNA-seq experiments with HISAT, StringTie and Ballgown. Nat Protoc.

[48] Huggett JF, Foy CA, Vladimir B, Kerry E, Garson JA, Ross H (2013). The digital MIQE guidelines: minimum information for publication of quantitative digital PCR experiments. Clin Chem.

[49] Ramakers C, Ruijter JM, Deprez RHL, Moorman AF (2003). Assumption-free analysis of quantitative real-time polymerase chain reaction (PCR) data. Neurosci Lett.

