# Supplementary material for: Genome-wide identification of ubiquitin proteasome subunits as superior reference genes for transcript normalization during receptacle development in strawberry cultivars
Source: BMC Genomics. 2021 Jan 28;22:88. doi: 10.1186/s12864-021-07393-9 (PMC7845027; doi:10.1186/s12864-021-07393-9)
Supplement: Supplementary file 1 — Additional file 1: Figure S1. The structure of strawberry fruit in ‘Ruegen’. Figure S2. Identification, phylogenetic and domain analyses of the Actin gene family in strawberry and Arabidopsis. Figure S3. Identification, phylogenetic and domain analyses of the GAPDH gene family in strawberry and Arabidopsis. Figure S4. Identification, phylogenetic and domain analyses of the Tubulin gene family in strawberry and Arabidopsis. Figure S5. Identification, phylogenetic and domain analyses of the EF1α gene family in strawberry and Arabidopsis. Figure S6. Identification, phylogenetic and domain analyses of the QUL gene family in strawberry and Arabidopsis. Figure S7. Identification, phylogenetic and domain analyses of the SWIB gene family in strawberry and Arabidopsis. Figure S8. Identification, phylogenetic and domain analyses of the FHA gene family in strawberry and Arabidopsis. Figure S9. Identification, phylogenetic and domain analyses of the UBC gene family in strawberry and Arabidopsis. Figure S10. Identification, phylogenetic and domain analyses of the AP2/ERF gene family in strawberry and Arabidopsis. Figure S11. Identification, and phylogenetic and domain analyses of the bZip gene family in strawberry and Arabidopsis. Figure S12. Identification, phylogenetic and domain analyses of the PDC gene family in strawberry and Arabidopsis. Figure S13. Identification, phylogenetic and domain analyses of the HISTH4 gene family in strawberry and Arabidopsis. Figure S14. Identification of qualified HKGs in strawberry receptacle development based on RNA-seq data. Figure S15. Schematic illustration of the cellular functions of nine “SRDS” RGs. Figure S16. Relative expression of candidate reference genes from RNA-seq data. Figure S17. Strawberry receptacle RNA sample quality assessment. Figure S18. Specificity assessment of RT-qPCR primers. Figure S19. Flow chart showing procedure for RT-qPCR analysis of candidate reference genes during strawberry receptacle development. Figure S20 [file 12864_2021_7393_MOESM1_ESM.zip › Supplemental Data.docx]

**Genome-wide Identification of Ubiquitin Proteasome Subunits as Superior Reference Genes for Transcript Normalization during Receptacle Development in Strawberry Cultivars**

Jianqing Chen^1,2†^*, Jinyu Zhou^1†^, Yanhong Hong^1†^, Zekun Li^1†^, Cheng xiangyu^1^, Aiying Zheng^1^, Yilin Zhang^1^, Juanjuan Song^1^, Guifeng Xie^1^, Changmei Chen^1^, Meng Yuan^1^, Tengyun Wang^1^, Qingxi Chen^1^*

† Equal contributors

* Joint corresponding authors

^1^College of Horticulture, Fujian Agriculture and Forestry University, Fuzhou 350002, China

^2^FAFU-UCR Joint Center for Horticultural Biology and Metabolomics, Haixia Institute of Science and Technology, Fujian Agriculture and Forestry University, Fuzhou 350002, China

*Correspondence and requests for materials should be addressed to: Jianqing Chen (Jianqingchen@fafu.edu.cn) or Qingxi Chen (cqx0246@fafu.edu.cn)

**Supporting data**

**Figure S1. The structure of strawberry fruit in** ‘Ruegen’**.**

In botanical terms, the fruit of strawberry is an aggregate fruit composed of multiple achenes on the surface of the juicy flesh, which is accessory tissue developed from the enlarged receptacle. Bar=1cm.

**Figure S2. Identification, phylogenetic and domain analyses of the *Actin* gene family in strawberry and *Arabidopsis*.**

Six *Actin* genes were identified from the *F. vesca* genome assembly v4. A phylogeny for Actin proteins from strawberry and *Arabidopsis* was reconstructed using the maximum likelihood (ML) algorithm. Domain structure analysis revealed that the Actin_domain (pfam: PF00022) was highly conserved and common to all Actin proteins of strawberry (red) and *Arabidopsis* (black). The conserved domains were obtained using the InterProScan tool. The blue labeled Actin protein (FaACT1; GenBank accession no. AB116565) is the most frequently used RG transcript for RT-qPCR in strawberry fruit studies.

**Figure S3. Identification, phylogenetic and domain analyses of the *GAPDH* gene family in strawberry and *Arabidopsis*.**

Six *GAPDH* genes were identified from the *F. vesca* genome assembly v4. A phylogeny for GAPDH proteins from strawberry and *Arabidopsis* was reconstructed using the ML algorithm. Domain structure analysis revealed that the Gp_dh_C (pfam: PF02800) and Gp_dh_N (pfam: PF00044) were highly conserved and common to all GAPDH proteins of strawberry (red) and *Arabidopsis* (black). The blue labeled GAPDH protein (FaGAPDH1, Genbank No. AB363963) is the most frequently used RG transcript for RT-qPCR in strawberry fruit studies.

**Figure S4. Identification, phylogenetic and domain analyses of the *Tubulin* gene family in strawberry and *Arabidopsis*.**

Thirteen *Tubulin* genes were identified from the *F. vesca* genome assembly v4. A phylogeny for Tubulin proteins from strawberry and *Arabidopsis* was reconstructed using the ML algorithm. Domain structure analysis revealed that the Tubulin (pfam: PF00091) and Tubulin_C (pfam: PF03953) were highly conserved and common to all Tubulin proteins of strawberry (red) and *Arabidopsis* (black). The conserved domains were obtained using the InterProScan tool. Notably, multiple members of tubulin as candidates were assessed in Amil-Ruiz’s study, so we did not label individual members from Amil-Ruiz’s study in here.

**Figure S5. Identification, phylogenetic and domain analyses of the *EF1α* gene family in strawberry and *Arabidopsis*.**

Three *EF1α* genes were identified from the *F. vesca* genome assembly v4. A phylogeny for EF1α proteins from strawberry and *Arabidopsis* was reconstructed using the ML algorithm. Domain structure analysis revealed that the GTP_EFTU (pfam: PF00009), GTP_EFTU D2 (pfam:PF03144) and GTP_EFTU D3 (pfam:PF03143) were highly conserved and common to all EF1α proteins of strawberry (red) and *Arabidopsis* (black). Notably, multiple members of EF1α as candidates were assessed in Amil-Ruiz’s study, so we did not label individual members from Amil-Ruiz’s study in here.

**Figure S6. Identification, phylogenetic and domain analyses of the *QUL* gene family in strawberry and *Arabidopsis*.**

Sixteen *QUL* genes were identified from the *F. vesca* genome assembly v4. A phylogeny for QUL proteins from strawberry and *Arabidopsis* was reconstructed using the ML algorithm. Domain structure analysis revealed that the Methytransf_29 (pfam: PF03141) was highly conserved and common to all QUL proteins of strawberry (red) and *Arabidopsis* (black). We labeled blue for the QUL member (FaMT1, the ID from *F. vesca* genome assembly v1: gene10517) from Amil-Ruiz’s study. FveQUL16 was homologous of FaMT1.

**Figure S7. Identification, phylogenetic and domain analyses of the *SWIB* gene family in strawberry and *Arabidopsis*.**

Nineteen *SWIB* genes were identified from the *F. vesca* genome assembly v4. A phylogeny for SWIB proteins from strawberry and *Arabidopsis* was reconstructed using the ML algorithm. Domain structure analysis revealed that the SWIB (pfam: PF02201) was highly conserved and common to all SWIB proteins of strawberry (red) and *Arabidopsis* (black). We labeled blue for the SWIB member (FaCHC, the ID from *F. vesca* genome assembly v1: gene25887) from Amil-Ruiz’s study. FveSWIB16 was homologous of FaCHC.

**Figure S8. Identification, phylogenetic and domain analyses of the *FHA* gene family in strawberry and *Arabidopsis*.**

Eight *FHA* genes were identified from the *F. vesca* genome assembly v4. A phylogeny for FHA proteins from strawberry and *Arabidopsis* was reconstructed using the ML algorithm. Domain structure analysis revealed that the FHA (pfam: PF00022) was highly conserved and common to all FHA proteins of strawberry (red) and *Arabidopsis* (black). We labeled blue for the FHA member (FaFHA1, the ID from *F. vesca* genome assembly v1: gene17571) from Amil-Ruiz’s study. FveFHA1 was homologous of FaFHA1.

**Figure S9. Identification, phylogenetic and domain analyses of the *UBC* gene family in strawberry and *Arabidopsis*.**

Forty-two *UBC* genes were identified from the *F. vesca* genome assembly v4. A phylogeny for UBC proteins from strawberry and *Arabidopsis* was reconstructed using the ML algorithm. Domain structure analysis revealed that the UQ_con (pfam: PF00179) was highly conserved and common to all UBC proteins of strawberry (red) and *Arabidopsis* (black). We labeled blue for the UBC member (FaUBC1, the ID from *F. vesca* genome assembly v1: gene08438) from Amil-Ruiz’s study. FveUBC50 was homologous of FaUBC1.

**Figure S10. Identification, phylogenetic and domain analyses of the *AP2/ERF* gene family in strawberry and *Arabidopsis*.**

One hundred-two *AP2*/*ERF* genes were identified from the *F. vesca* genome assembly v4. A phylogeny for ERF proteins from strawberry and *Arabidopsis* was reconstructed using the ML algorithm. Domain structure analysis revealed AP2 (pfam: PF00847) was highly conserved and common to all ERF proteins of strawberry (red) and *Arabidopsis* (black). We labeled blue for the ERF member (FaTIM1, the ID from *F. vesca* genome assembly v1: gene17570) from Amil-Ruiz’s study. FveERF102 was homologous of FaTIM1.

**Figure S11. Identification, and phylogenetic and domain analyses of the *bZip* gene family in strawberry and *Arabidopsis*.**

Fifty-four *bZip* genes were identified from the *F. vesca* genome assembly v4. A phylogeny for bZip proteins from strawberry and *Arabidopsis* was reconstructed using the ML algorithm. Domain structure analysis revealed bZip_1 (pfam: PF00170) or bZip_2 (pfam: PF07716) were highly conserved and common to all bZip proteins of strawberry (red) and *Arabidopsis* (black). We labeled blue for the bZip member (FabZip1, the ID from *F. vesca* genome assembly v1: gene17796) from Amil-Ruiz’s study. FvebZIP20 was homologous of FabZip1.

**Figure S12. Identification, and phylogenetic and domain analyses of the *PDC* gene family in strawberry and *Arabidopsis*.**

Three *PDC* genes were identified from the *F. vesca* genome assembly v4. A phylogeny for PDC proteins from strawberry and *Arabidopsis* was reconstructed using the ML algorithm. Domain structure analysis revealed TPP_enzyme_N (pfam: PF02776), TPP_enzyme_M (pfam: PF00205), and TPP_enzyme_C (pfam: PF02775) were highly conserved and common to all PDC proteins of strawberry (red) and *Arabidopsis* (black). We labeled blue for the PDC member (FaPIRUV) from Galli’s study. FvePDC1 was homologous of FaPIRUV.

**Figure S13. Identification, and phylogenetic and domain analyses of the *HISTH4* gene family in strawberry and *Arabidopsis*.**

Eight *HISTH4* genes were identified from the *F. vesca* genome assembly v4. A phylogeny for HISTH4 proteins from strawberry and *Arabidopsis* was reconstructed using the ML algorithm. Domain structure analysis revealed CENP-T_C (pfam: PF15511) is highly conserved and common to all HISTH4 proteins of strawberry (red) and *Arabidopsis* (black). We labeled blue for the HISTH4 member (FaHISTH4) from Galli’s study. FveHISTH4.1 was homologous of FaHISTH4.

**Figure S14. Identification of qualified HKGs in strawberry receptacle development based on RNA-seq data.**

To discover additional qualified HKGs during receptacle development, we adopted a screening procedure with cut-off values for CV ≤ 0.2 and FPKM ≥ 100 in nine RNA-seq data sets that include receptacle development experiments in strawberry. Statistical analysis of CV and FPKM values of ten HKG families identified from the nine RNA-seq data sets. The CV analysis is shown on the left side of the figure, and the FPKM analysis is shown on the right side of the figure. Each data point in the box-plot is derived from one RNA-seq data set. The horizontal line in the box represents the median. The red dashed lines indicate the cut-off values. The red background indicates qualified HKGs. The blue background indicates HKGs homologous of the ones in Amil-Ruiz’s study. The pink background indicates HKGs meet simultaneously the conditions of qualified and homologous of the ones in Amil-Ruiz’s study.

**Figure S15. Schematic illustration of the cellular functions of nine “SRDS” RGs.**

Seven of the novel RGs were associated with the ubiquitin proteasome system (UPS) proteolytic pathway. These candidate genes covered most of the processes of the UPS, including proteasome assembly, ubiquitination, energy supply, and ubiquitin recycling. *RPN5A* and *RPT6A* encode subunits for assembly of the 26S proteasome; *UBC12* and *ASK1* synergistically target the ubiquitin to degrade protein; *VPS34* is a subunit of ESCRT-III for profoundly shaping signal transduction, which is based on the degradative sorting of ubiquitinated membrane proteins and deubiquitylation; *ATPD* and *ATPE*, which encode two subunits of F_0_F_1_-ATP synthase, produce abundant ATP to ensure normal UPS operation. Two additional uncorrelated UPS genes, *AKR2B* and *YLS8*, were identified as “SRDS” RGs. *AKR2B* is a molecular chaperone that delivers target membrane-associated proteins to their membrane; *YLS8* encodes a mitosis protein, but its precise function is unknown.

**Figure S16. Relative expression of candidate reference genes from RNA-seq data.**

Twenty-two candidate RGs were identified from 80 RNA-seq libraries including data for strawberry receptacle development. Relative expression levels per gene were derived by dividing the expression value per library by the average expression level calculated across each RNA-seq experiment.

**Figure S17 Strawberry receptacle RNA sample quality assessment.**

**(a)** Integrity of RNA was confirmed by agarose gel analysis. The ribosomal RNA bands are clearly visible indicating that the RNA is intact. **(b)** RNA quality was further assessed by determining the OD 260/280 ratio using a Nanodrop tool. All values are close to 2 indicating good RNA quality. For detailed description of strawberry fruit samples used, see Figure 4.

**Figure S18. Specificity assessment of RT-qPCR primers.**

**(a)** Melting curve analysis of the specificity of primer pairs of candidate RGs in RT-qPCR. **(b)** Electrophoretic analysis of PCR product after RT-qPCR on a 2.5% agarose gel showed that single band with predict product size. Both analysis confirm the specificity of the primer pairs used in this study.

**Figure S19. Flow chart showing procedure for RT-qPCR analysis of candidate reference genes during strawberry receptacle development.**

Critical threshold (CT) values for candidate RGs were calculated by different algorithms. The efficiency of each primer for a candidate RG was evaluated before use. The distribution of the CT data was checked by means of a box plot. To evaluate the expression stability of genes, gene-stability measure (M), stability (Stab), coefficient of variation (CV), and standard deviation (SD) values were calculated using geNorm, NormFinder, BestKeeper, and Delta CT, respectively. The results of each stability estimation were merged using RankAggreg to generate a consensus ranking of the RGs. The pairwise variation (PV, *V_n_*/*V_n_*_+1_), as a model-based approach, was calculated to determine the optimal number of RGs for normalization of gene expression in receptacle development. Finally, considering the above assessments, we determined the optimal RGs.

**Figure S20. Expression stability of candidate reference genes of ‘Ruegen’ analyzed by RT-qPCR**

To evaluate the expression stability of the RGs, gene-stability M, stability, CV, and SD values were calculated using geNorm (**a**), BestKeeper (**c**), NormFinder (**d**) and Delta CT (**e**). A lower value indicates greater stability of expression. The RankAggreg package for R was employed to merge the stability measurements obtained from the four tools using a Monte Carlo algorithm and to establish a consensus ranking of the RGs (**f**). The pairwise variation (*V_n_*/*V_n_*_+1_) was calculated to determine the optimal number of RGs for normalization of gene expression (**b**).

**Figure S21. Expression stability of candidate reference genes of ‘Monterey’ analyzed by RT-qPCR**

To evaluate the expression stability of the RGs, gene-stability M, stability, CV, and SD values were calculated using geNorm (**a**), BestKeeper (**c**), NormFinder (**d**) and Delta CT (**e**). A lower value indicates greater stability of expression. The RankAggreg package for R was employed to merge the stability measurements obtained from the four tools using a Monte Carlo algorithm and to establish a consensus ranking of the RGs (**f**). The pairwise variation (*V_n_*/*V_n_*_+1_) was calculated to determine the optimal number of RGs for normalization of gene expression (**b**).

**Table S1. Description of 80 samples with receptacle development for RNA-seq in strawberry.**

**Table S2. The information of HKG families in strawberry and *Arabidopsis*.**
