# Supplementary material for: Genome-wide identification of ubiquitin proteasome subunits as superior reference genes for transcript normalization during receptacle development in strawberry cultivars
Source: BMC Genomics. 2021 Jan 28;22:88. doi: 10.1186/s12864-021-07393-9 (PMC7845027; doi:10.1186/s12864-021-07393-9)
Supplement: Supplementary file 1 — Additional file 1: Figure S1. The structure of strawberry fruit in ‘Ruegen’. Figure S2. Identification, phylogenetic and domain analyses of the Actin gene family in strawberry and Arabidopsis. Figure S3. Identification, phylogenetic and domain analyses of the GAPDH gene family in strawberry and Arabidopsis. Figure S4. Identification, phylogenetic and domain analyses of the Tubulin gene family in strawberry and Arabidopsis. Figure S5. Identification, phylogenetic and domain analyses of the EF1α gene family in strawberry and Arabidopsis. Figure S6. Identification, phylogenetic and domain analyses of the QUL gene family in strawberry and Arabidopsis. Figure S7. Identification, phylogenetic and domain analyses of the SWIB gene family in strawberry and Arabidopsis. Figure S8. Identification, phylogenetic and domain analyses of the FHA gene family in strawberry and Arabidopsis. Figure S9. Identification, phylogenetic and domain analyses of the UBC gene family in strawberry and Arabidopsis. Figure S10. Identification, phylogenetic and domain analyses of the AP2/ERF gene family in strawberry and Arabidopsis. Figure S11. Identification, and phylogenetic and domain analyses of the bZip gene family in strawberry and Arabidopsis. Figure S12. Identification, phylogenetic and domain analyses of the PDC gene family in strawberry and Arabidopsis. Figure S13. Identification, phylogenetic and domain analyses of the HISTH4 gene family in strawberry and Arabidopsis. Figure S14. Identification of qualified HKGs in strawberry receptacle development based on RNA-seq data. Figure S15. Schematic illustration of the cellular functions of nine “SRDS” RGs. Figure S16. Relative expression of candidate reference genes from RNA-seq data. Figure S17. Strawberry receptacle RNA sample quality assessment. Figure S18. Specificity assessment of RT-qPCR primers. Figure S19. Flow chart showing procedure for RT-qPCR analysis of candidate reference genes during strawberry receptacle development. Figure S20 [file 12864_2021_7393_MOESM1_ESM.zip › Supplemental figure.pptx]

## Slide 1
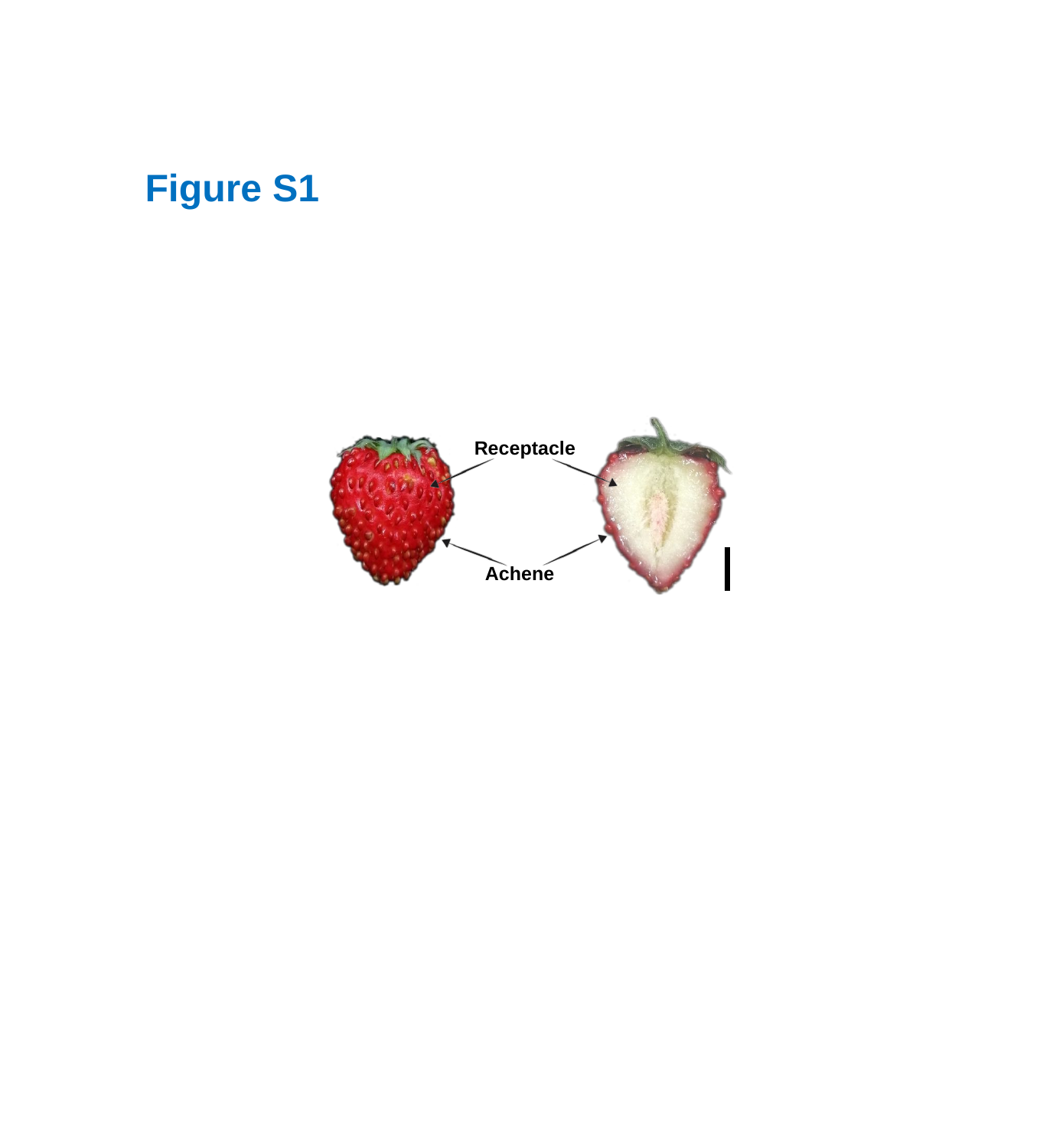

Figure S1
Receptacle
Achene

## Slide 2
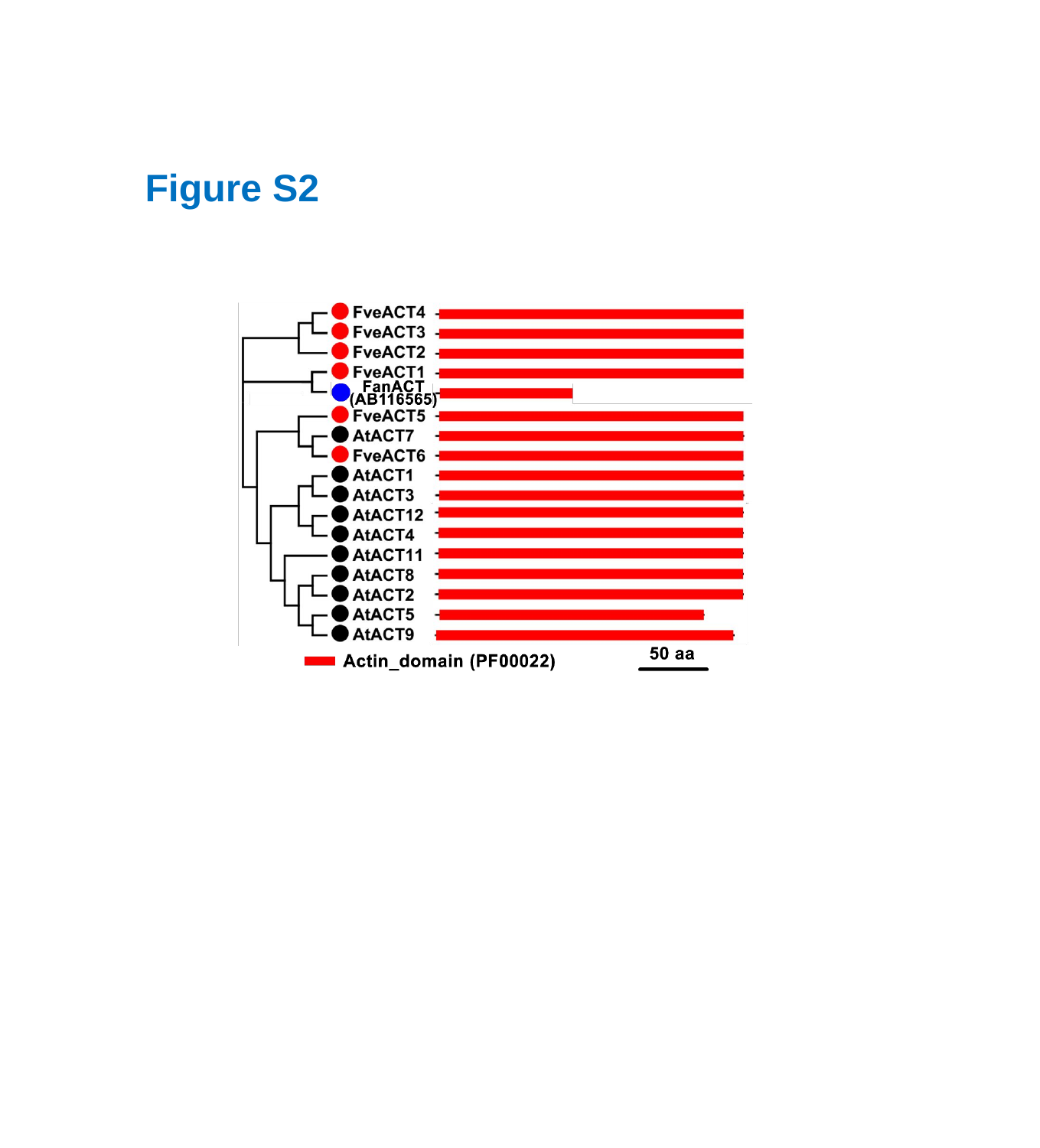

Figure S2

## Slide 3
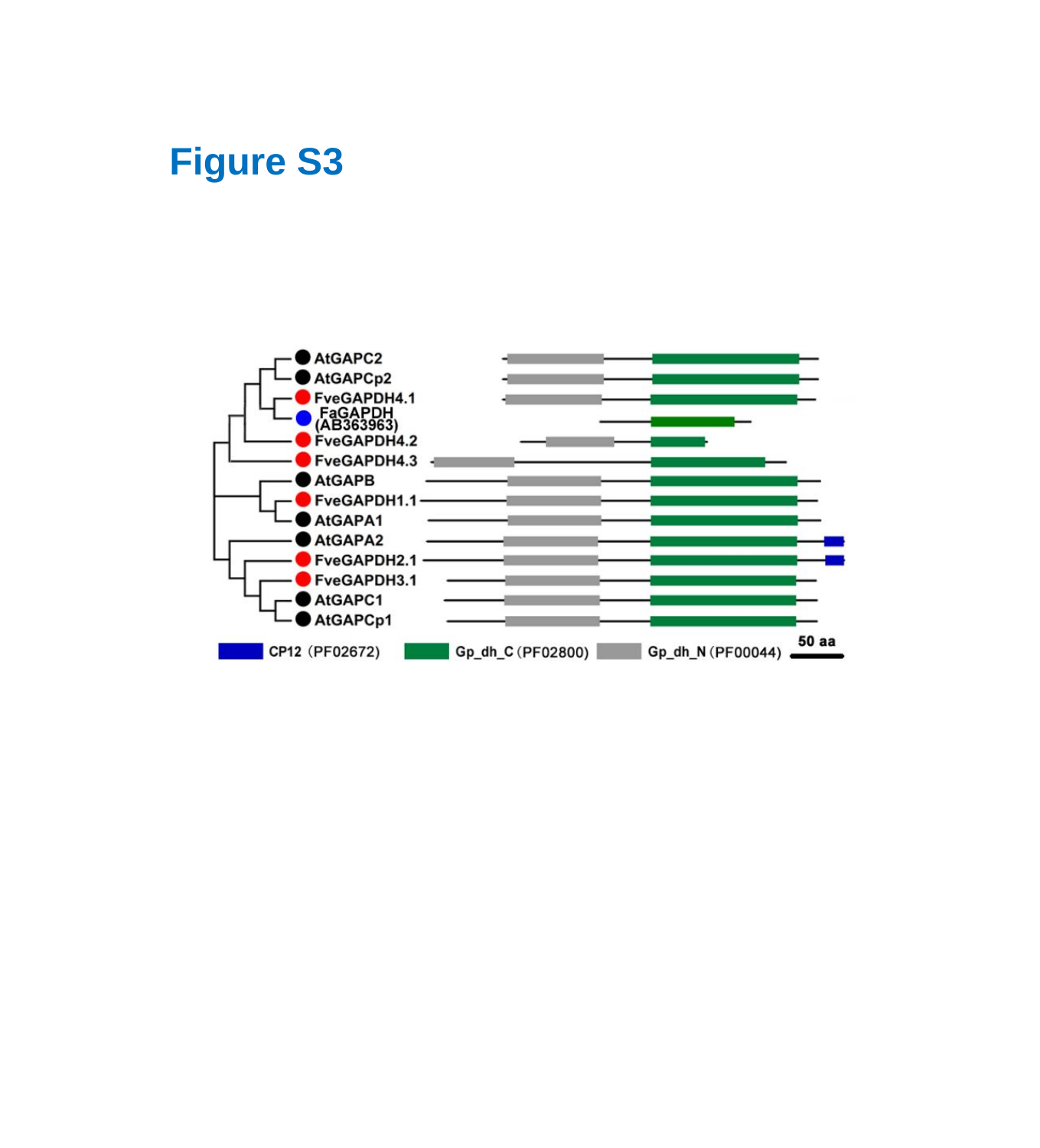

Figure S3

## Slide 4
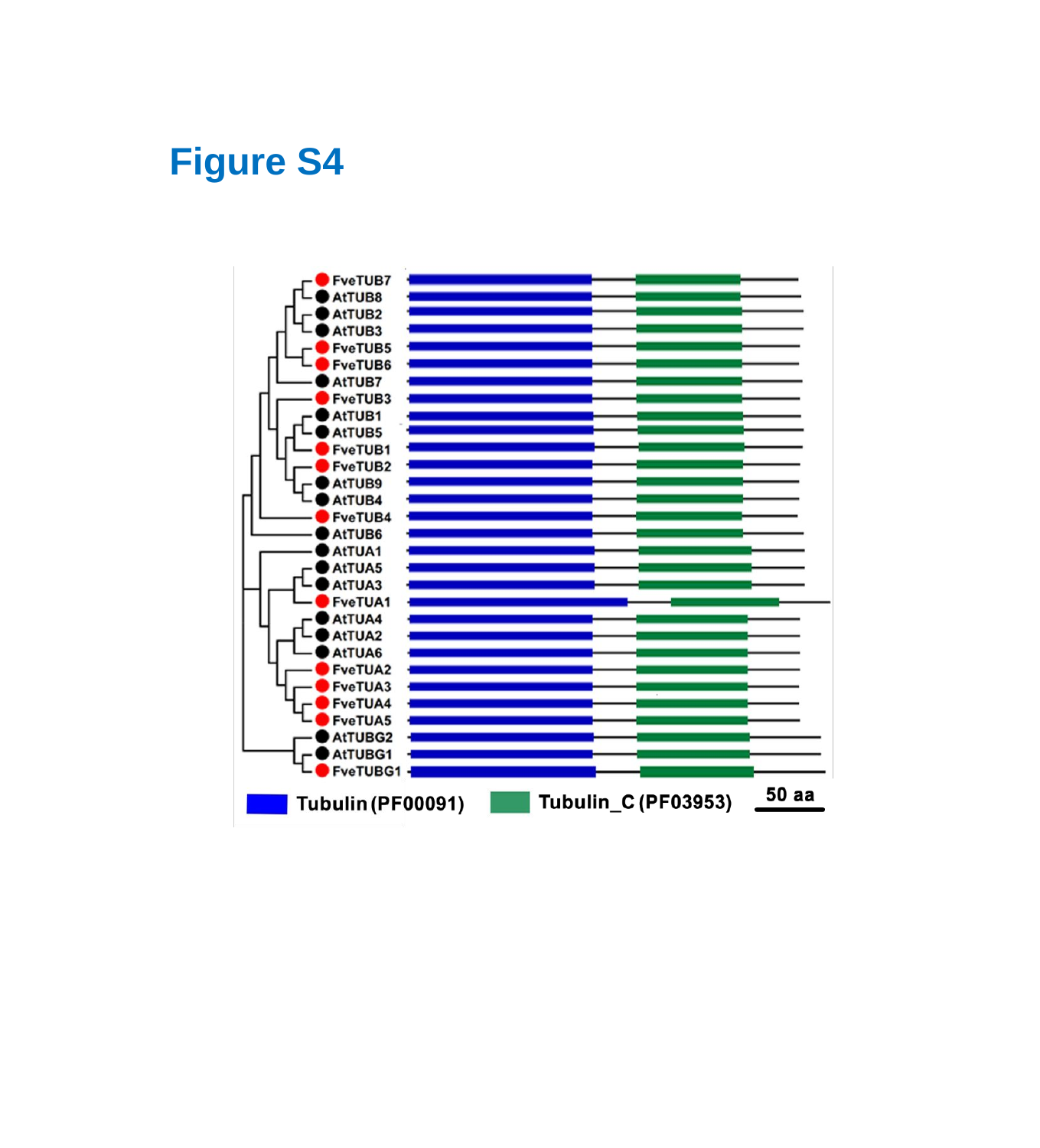

Figure S4

## Slide 5
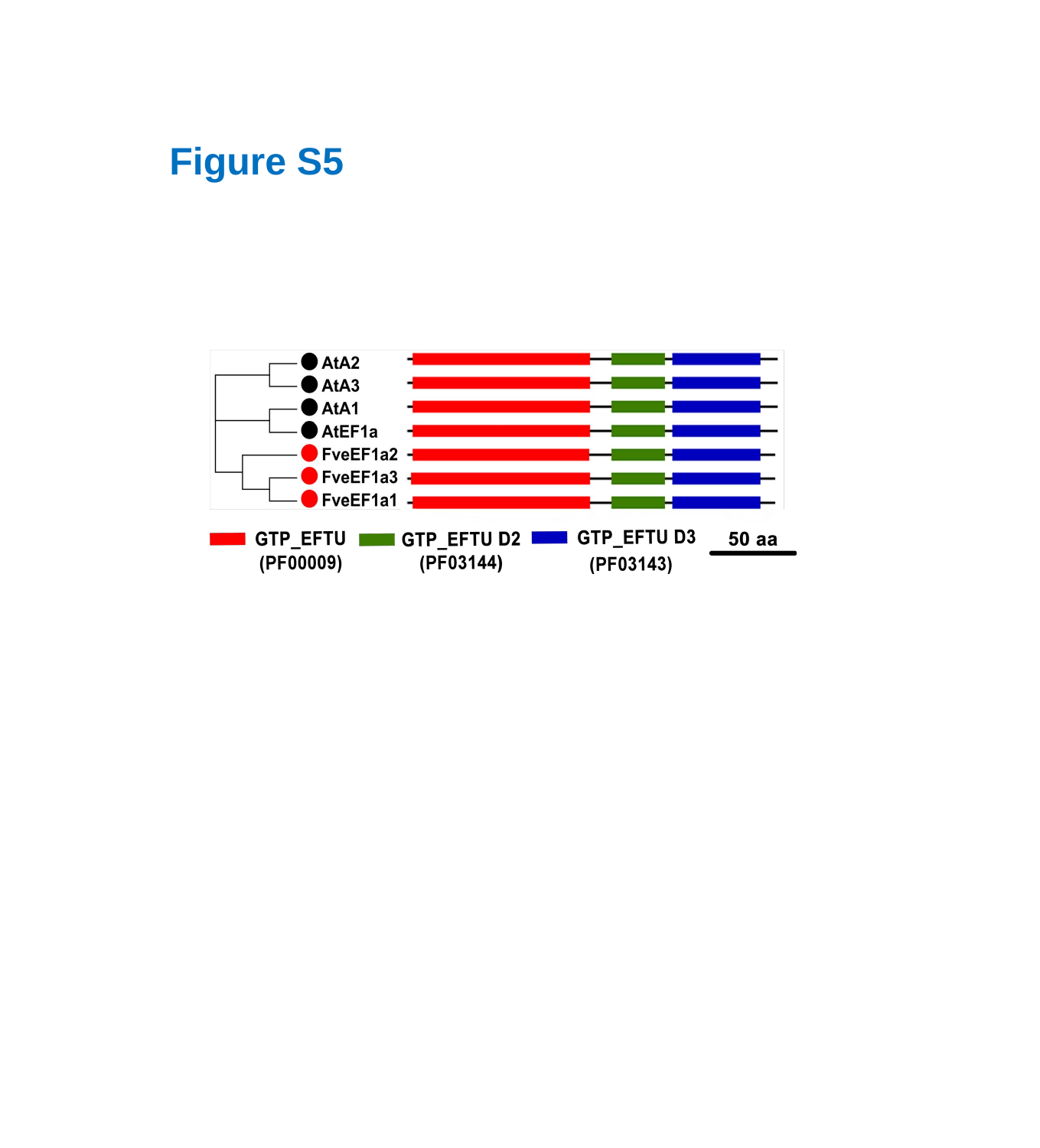

Figure S5

## Slide 6
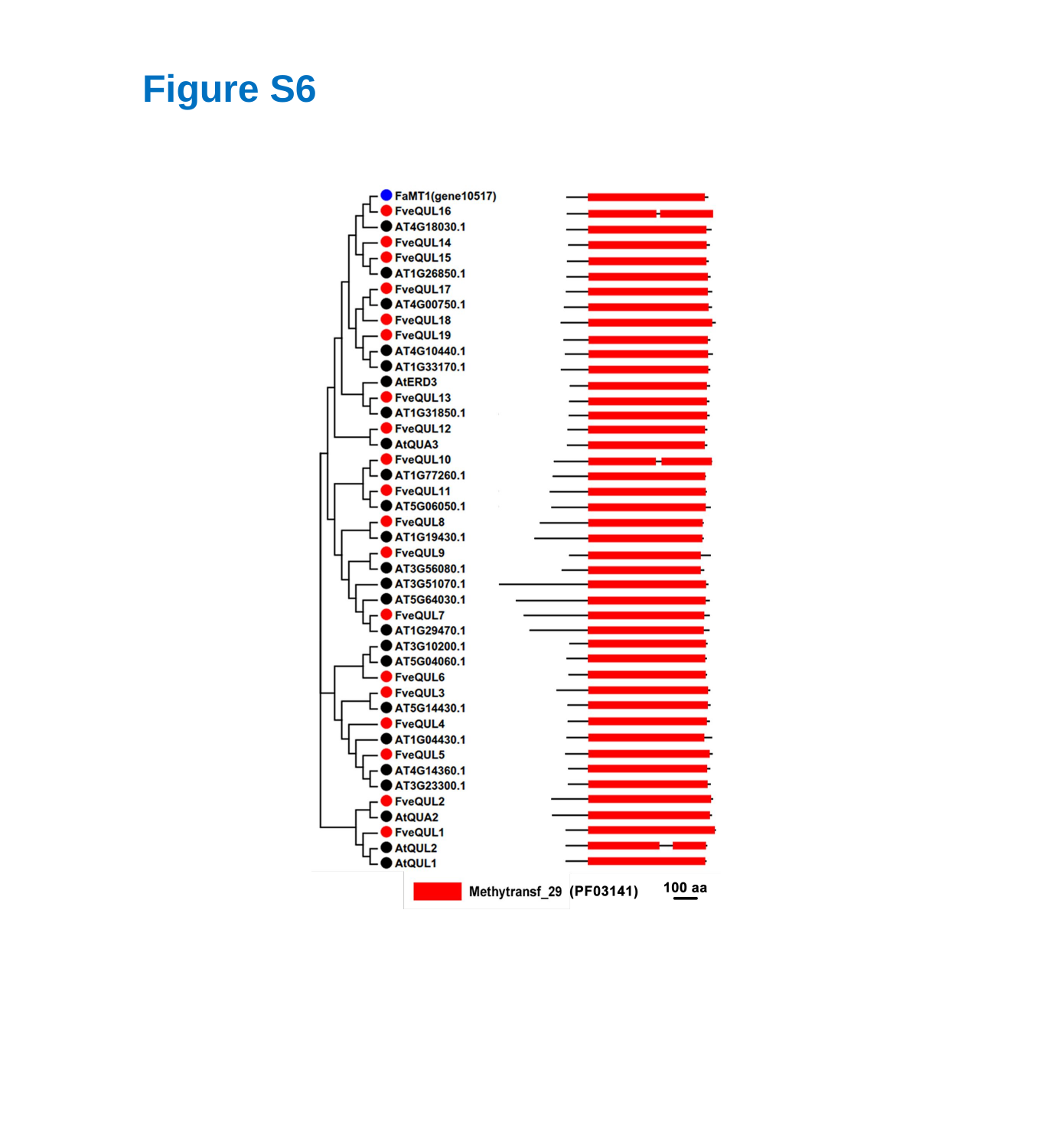

Figure S6

## Slide 7
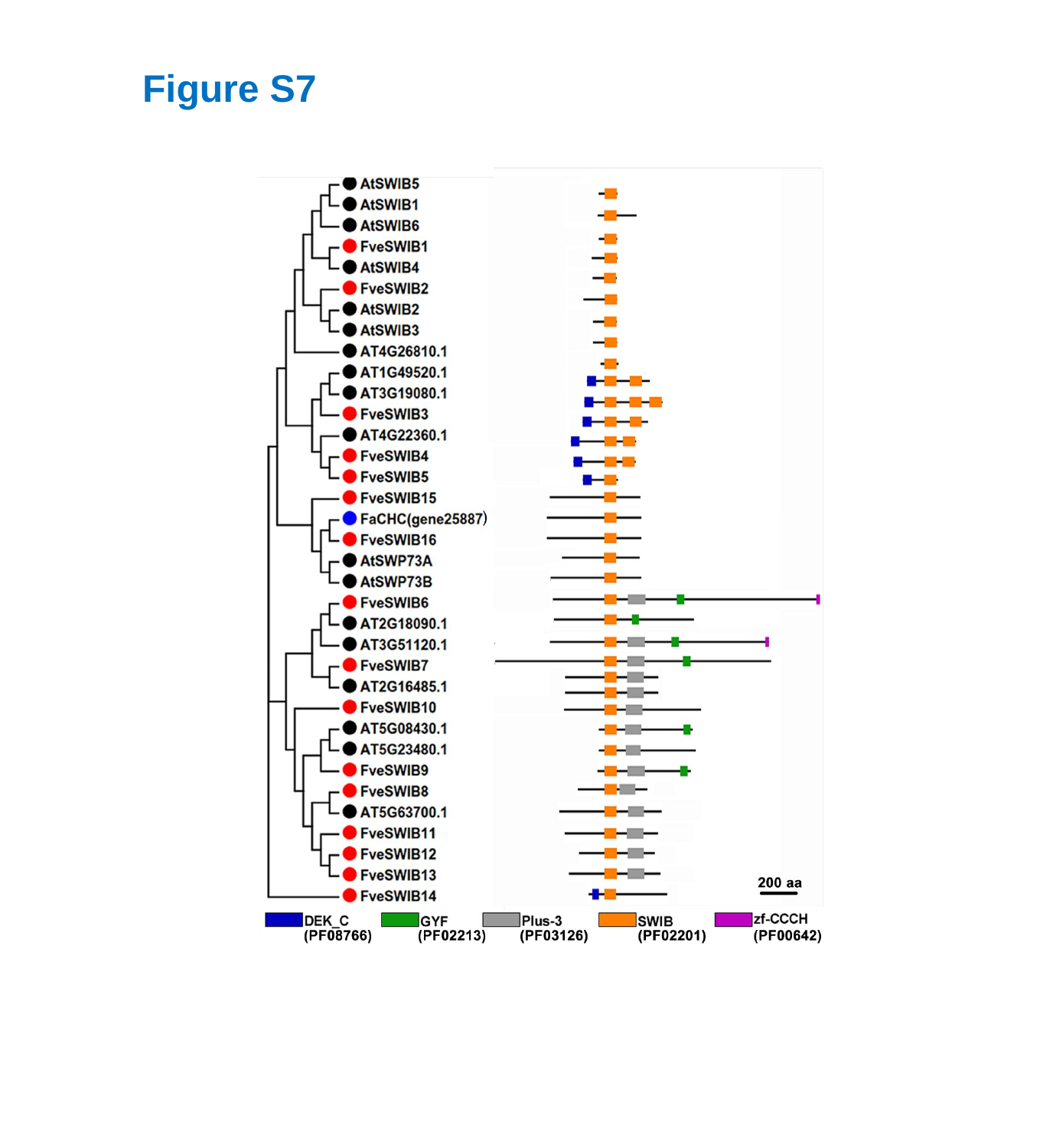

Figure S7

## Slide 8
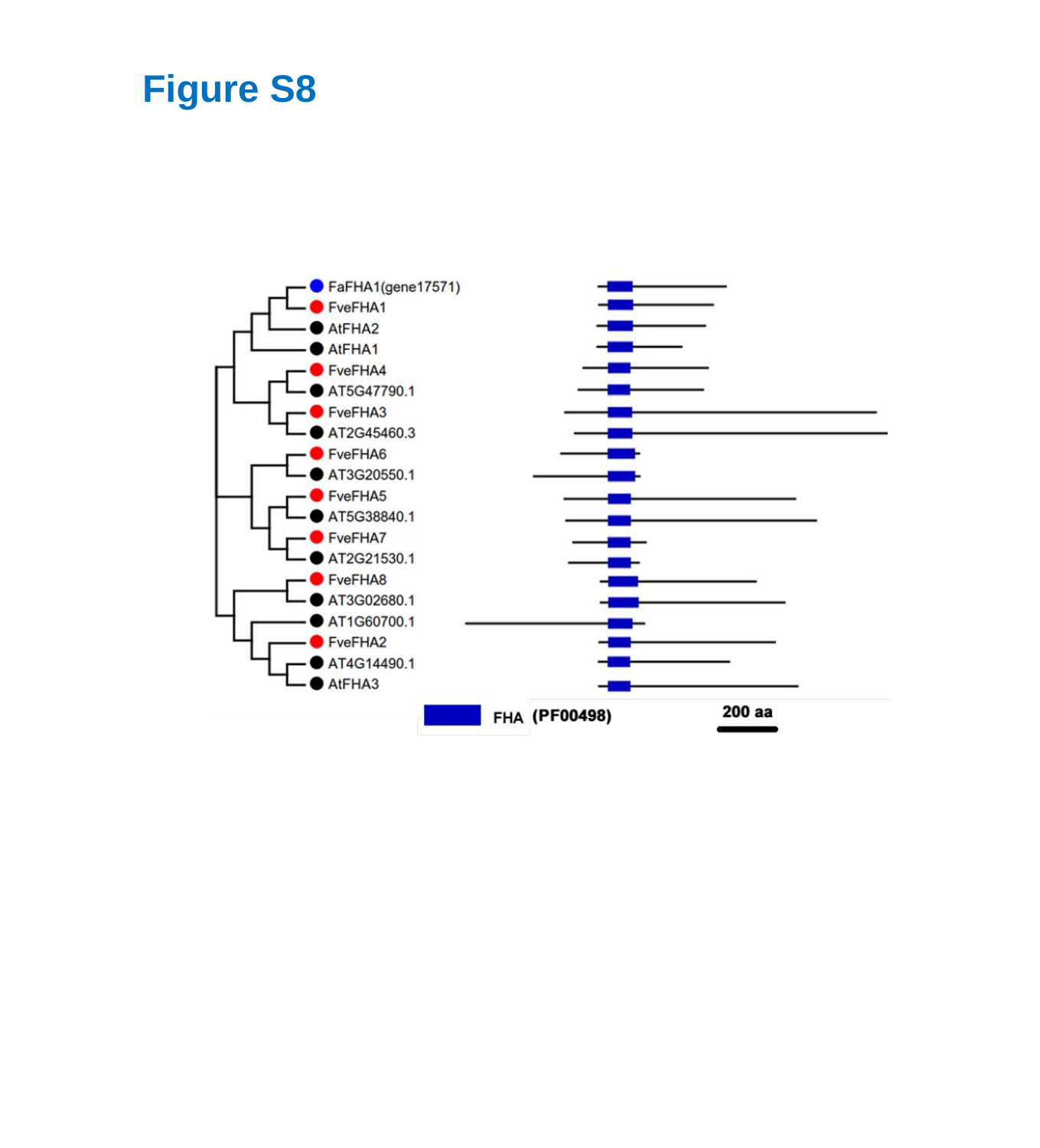

Figure S8

## Slide 9
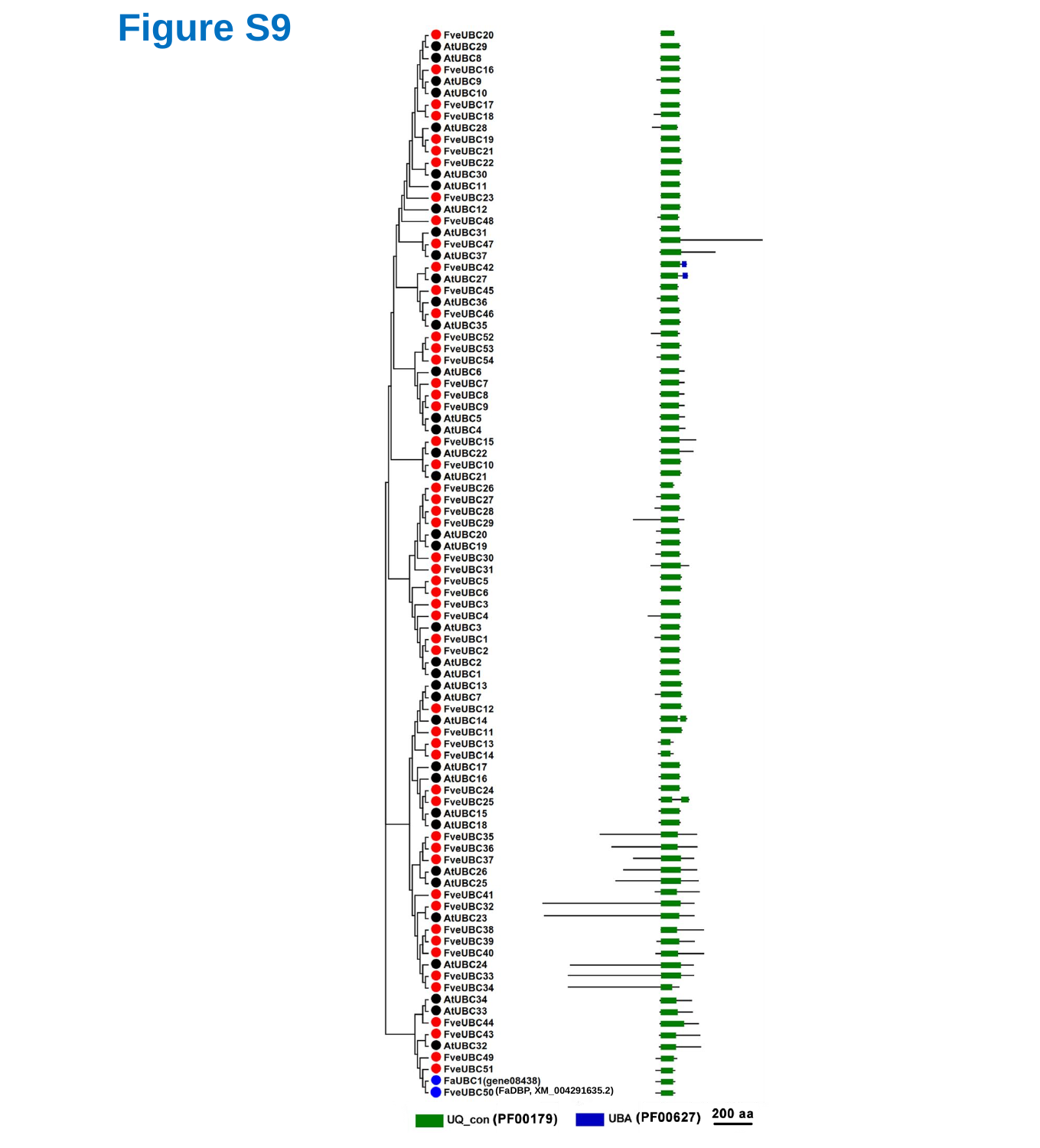

Figure S9
(FaDBP, XM_004291635.2)

## Slide 10
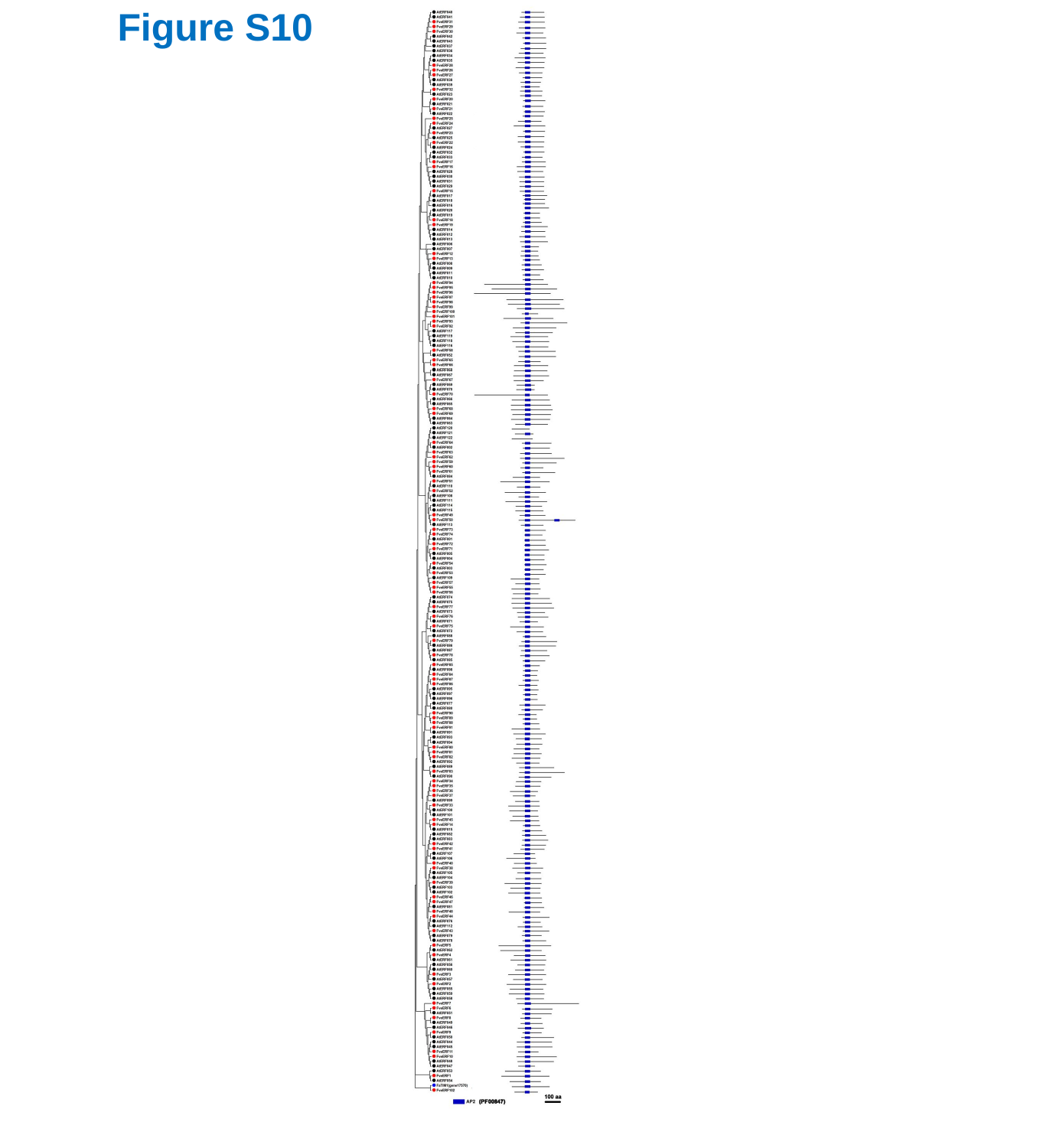

Figure S10

## Slide 11
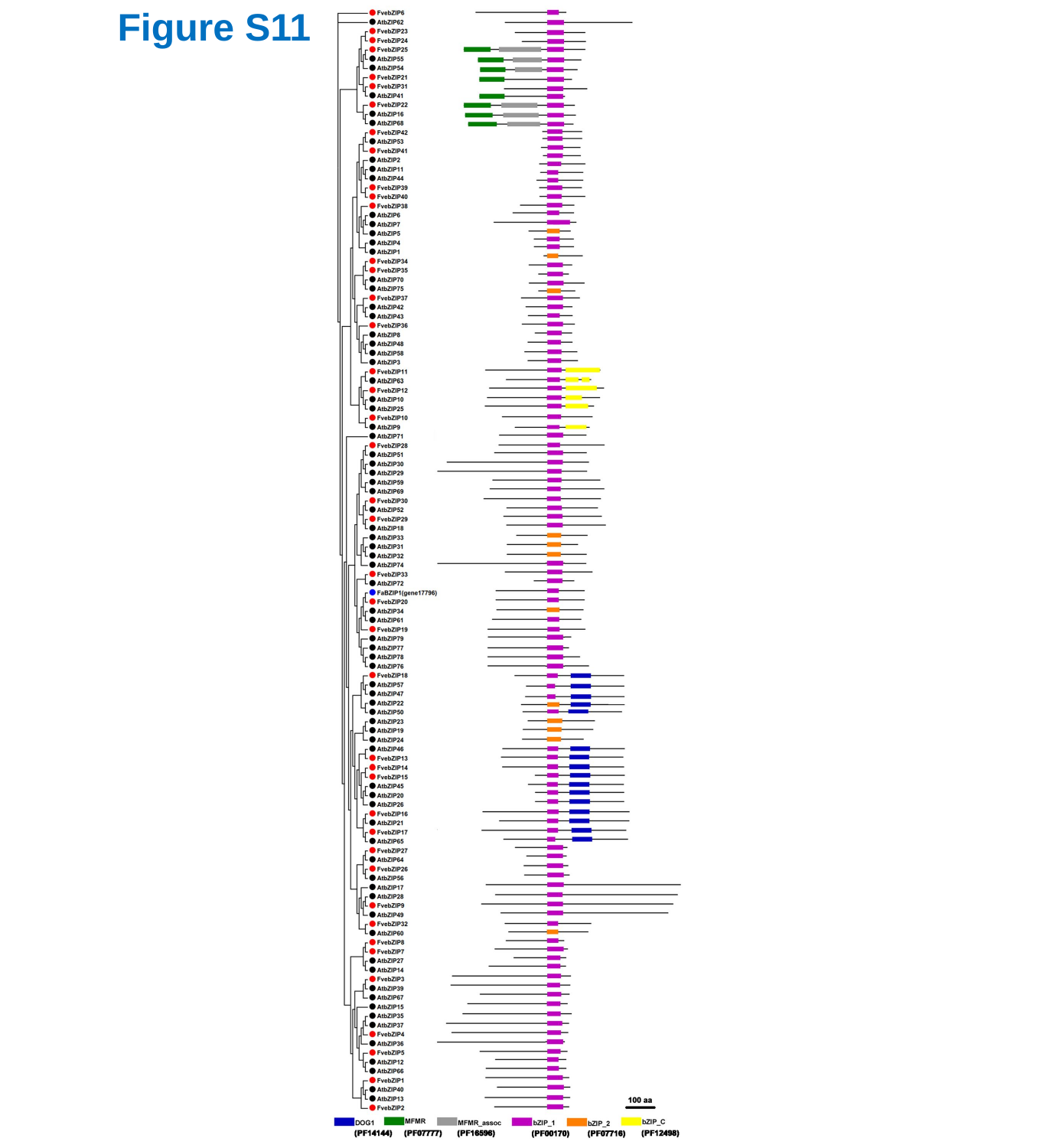

Figure S11

## Slide 12
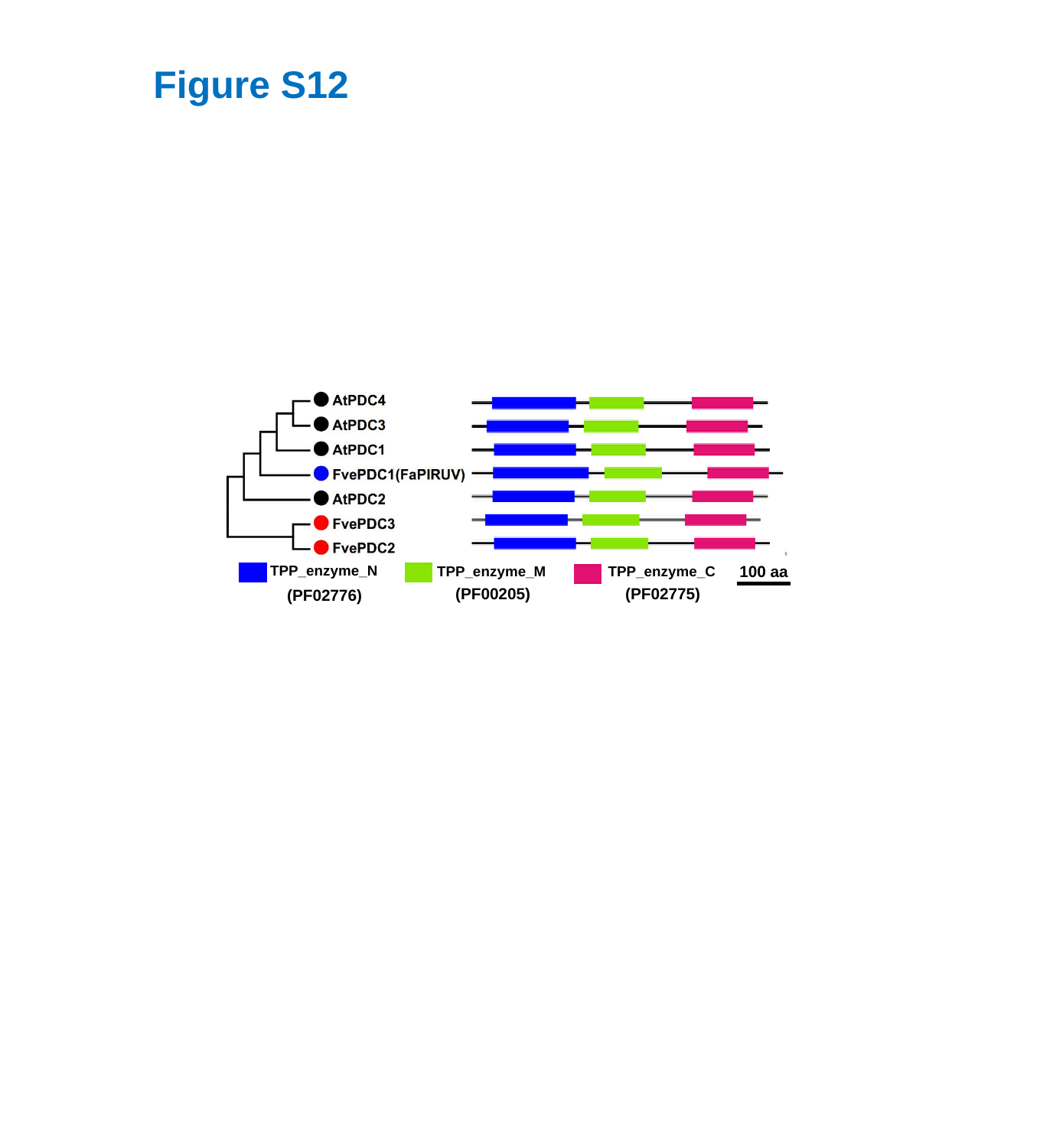

Figure S12
TPP_enzyme_N
100 aa
TPP_enzyme_M
TPP_enzyme_C
(PF00205)
(PF02775)
(PF02776)

## Slide 13
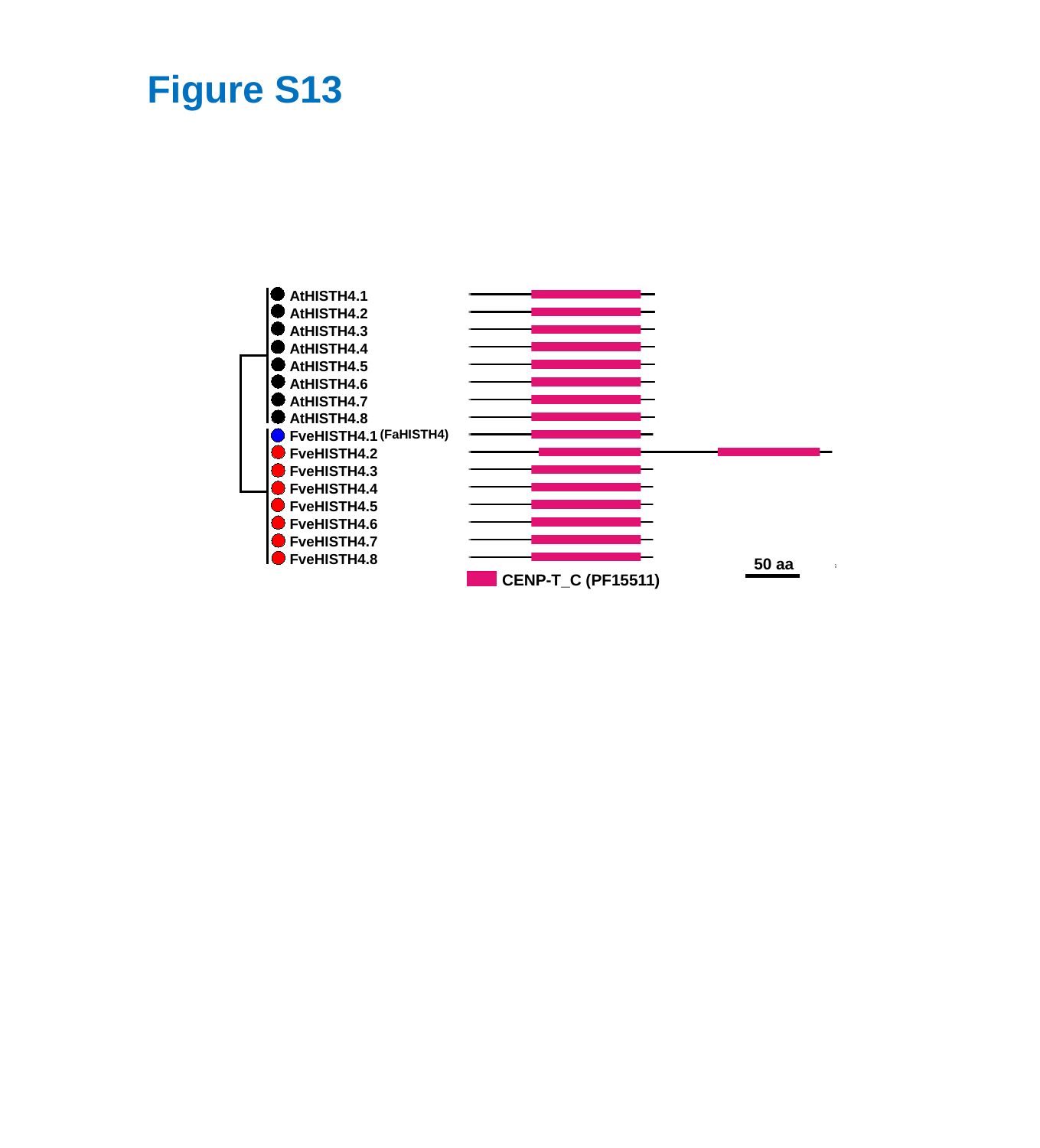

Figure S13
AtHISTH4.1
AtHISTH4.2
AtHISTH4.3
AtHISTH4.4
AtHISTH4.5
AtHISTH4.6
AtHISTH4.7
AtHISTH4.8
FveHISTH4.1
FveHISTH4.2
FveHISTH4.3
FveHISTH4.4
FveHISTH4.5
FveHISTH4.6
FveHISTH4.7
FveHISTH4.8
(FaHISTH4)
50 aa
CENP-T_C (PF15511)

## Slide 14
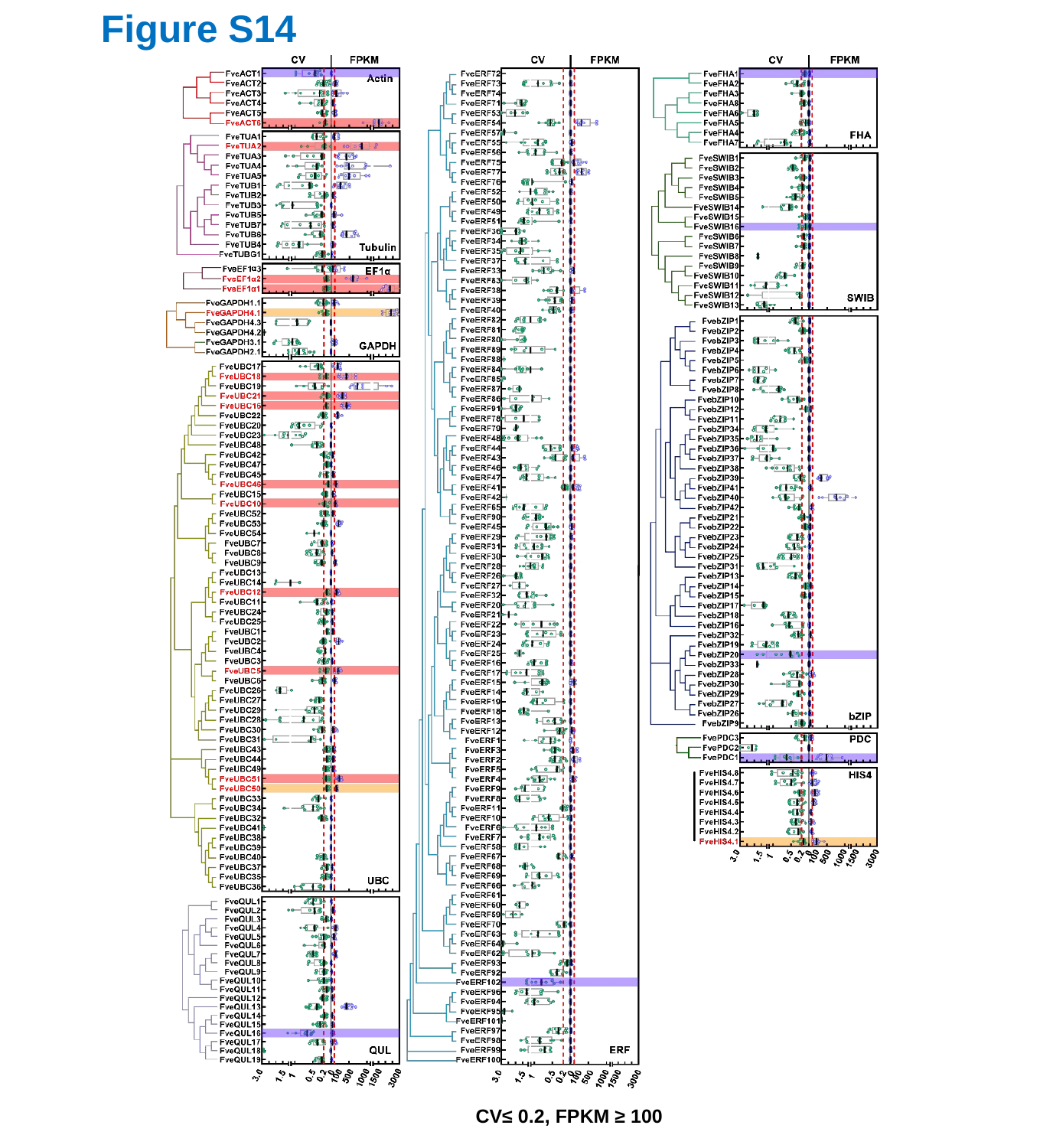

Figure S14
CV≤ 0.2, FPKM ≥ 100

## Slide 15
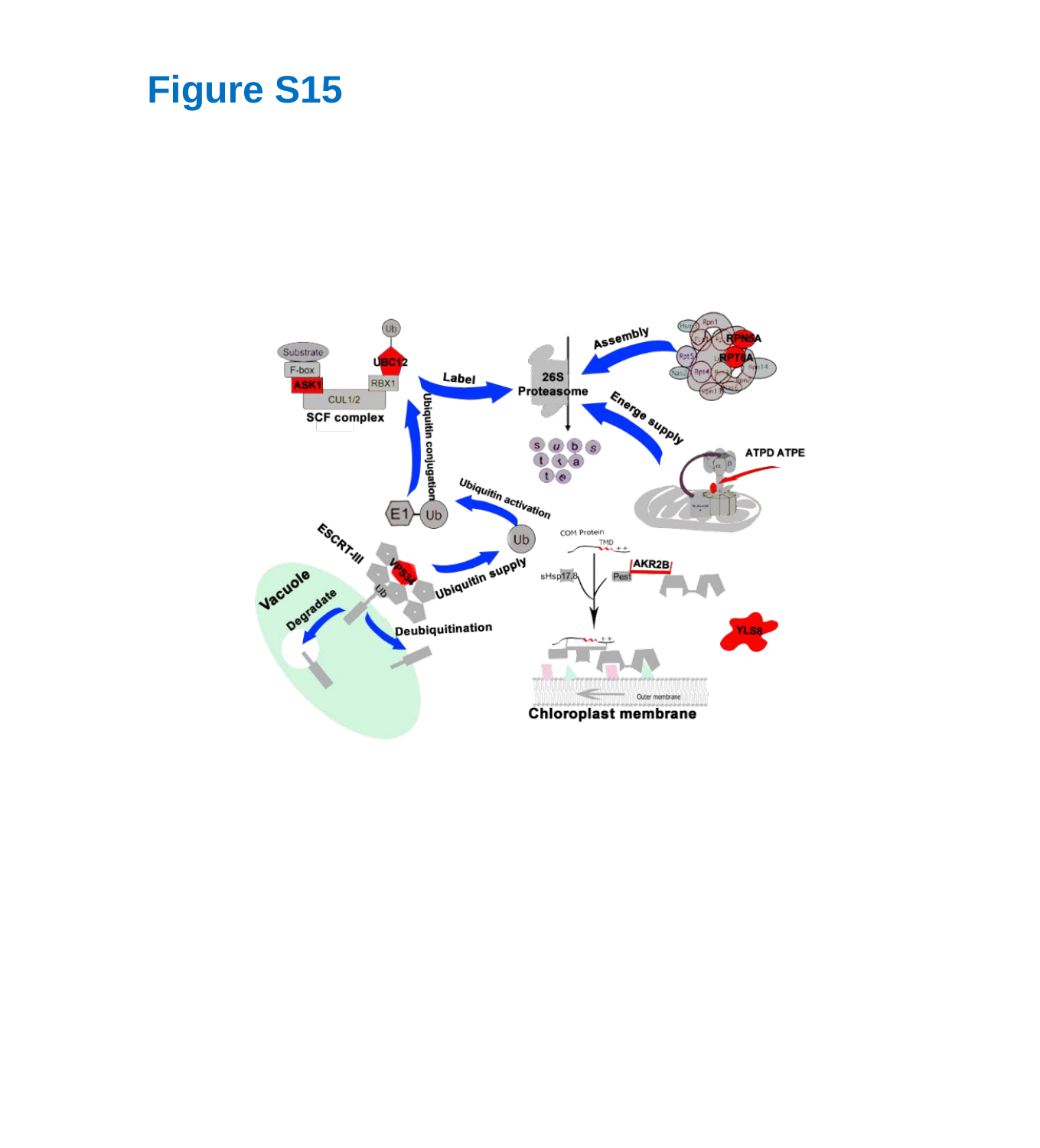

Figure S15

## Slide 16
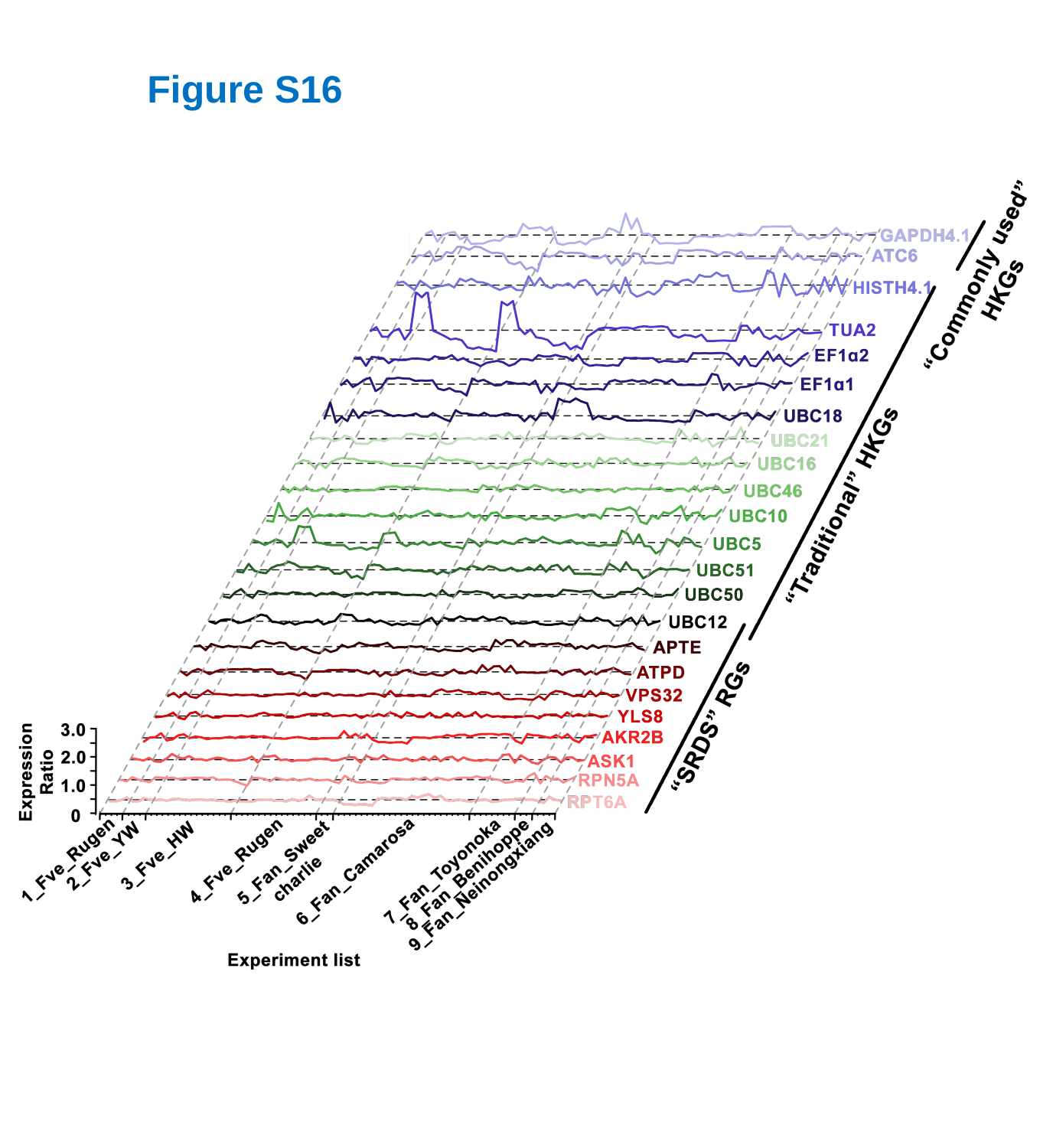

Figure S16

## Slide 17
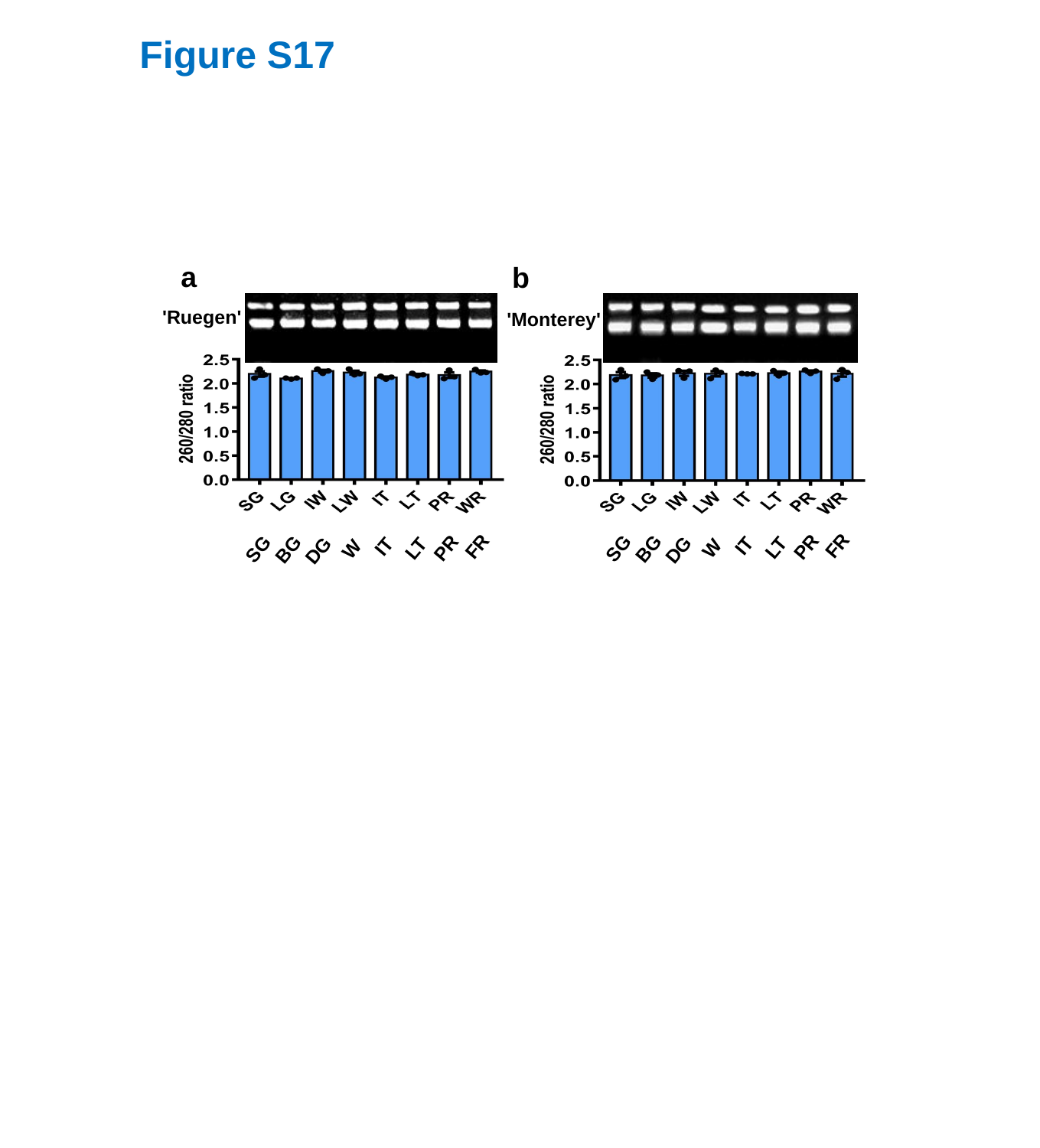

Figure S17
a
b
'Ruegen'
'Monterey'
IT
W
FR
LT
PR
SG
BG
DG
IT
W
FR
LT
PR
SG
BG
DG

## Slide 18
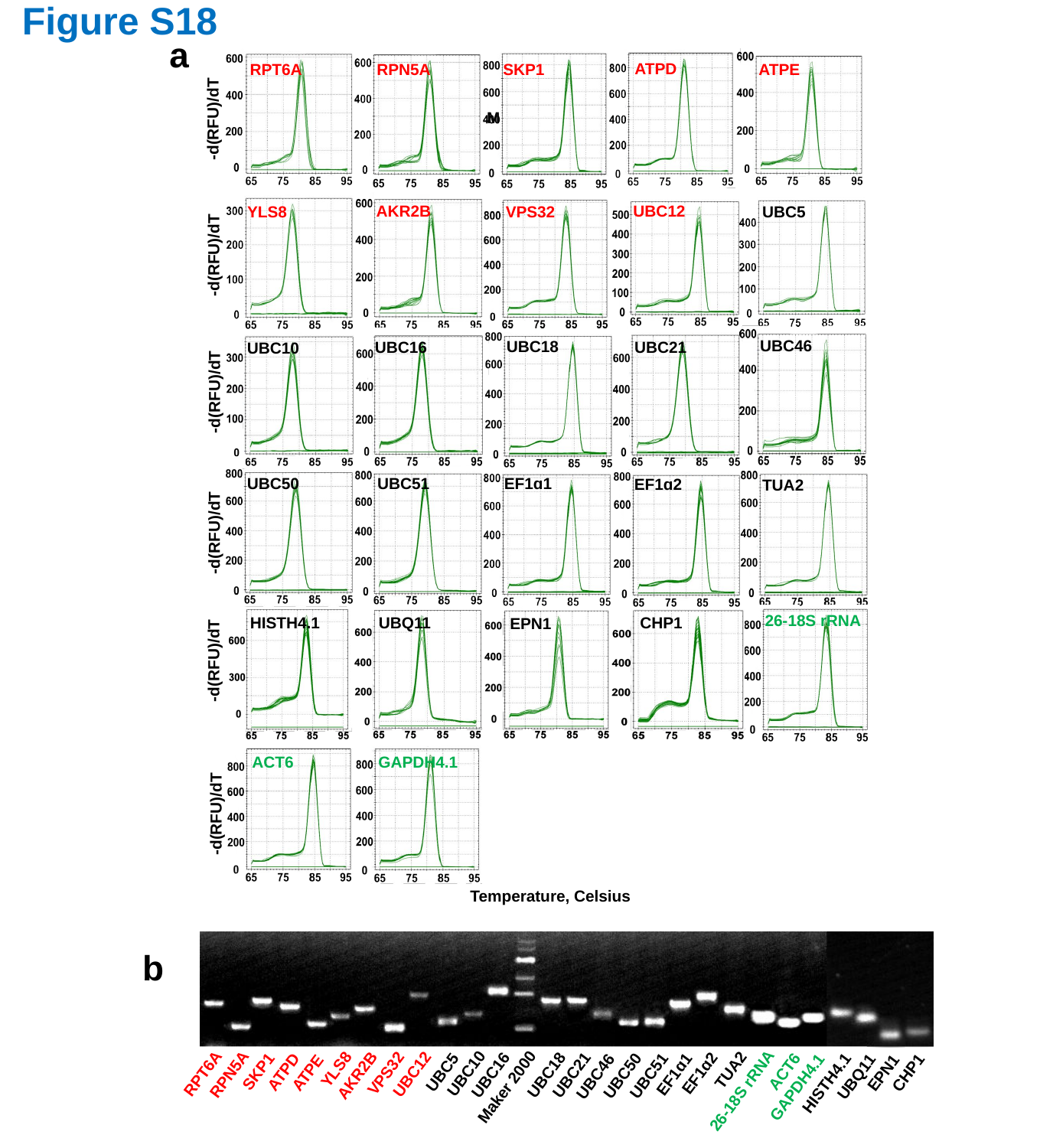

Figure S18
a
RPT6A
ATPE
ATPD
RPN5A
SKP1
UBC12
YLS8
AKR2B
UBC5
VPS32
UBC46
UBC16
UBC21
UBC18
UBC10
UBC50
EF1ɑ1
UBC51
EF1ɑ2
TUA2
26-18S rRNA
HISTH4.1
UBQ11
CHP1
EPN1
ACT6
GAPDH4.1
-d(RFU)/dT
-d(RFU)/dT
-d(RFU)/dT
-d(RFU)/dT
-d(RFU)/dT
-d(RFU)/dT
Temperature, Celsius
Melt Peak
b
RPT6A
TUA2
UBC21
YLS8
ACT6
ATPD
ATPE
UBC5
EF1ɑ2
VPS32
EF1ɑ1
UBC10
UBC12
RPN5A
UBC16
UBC18
UBC51
UBC50
AKR2B
UBC46
GAPDH4.1
26-18S rRNA
SKP1
Maker 2000
EPN1
CHP1
UBQ11
HISTH4.1

## Slide 19
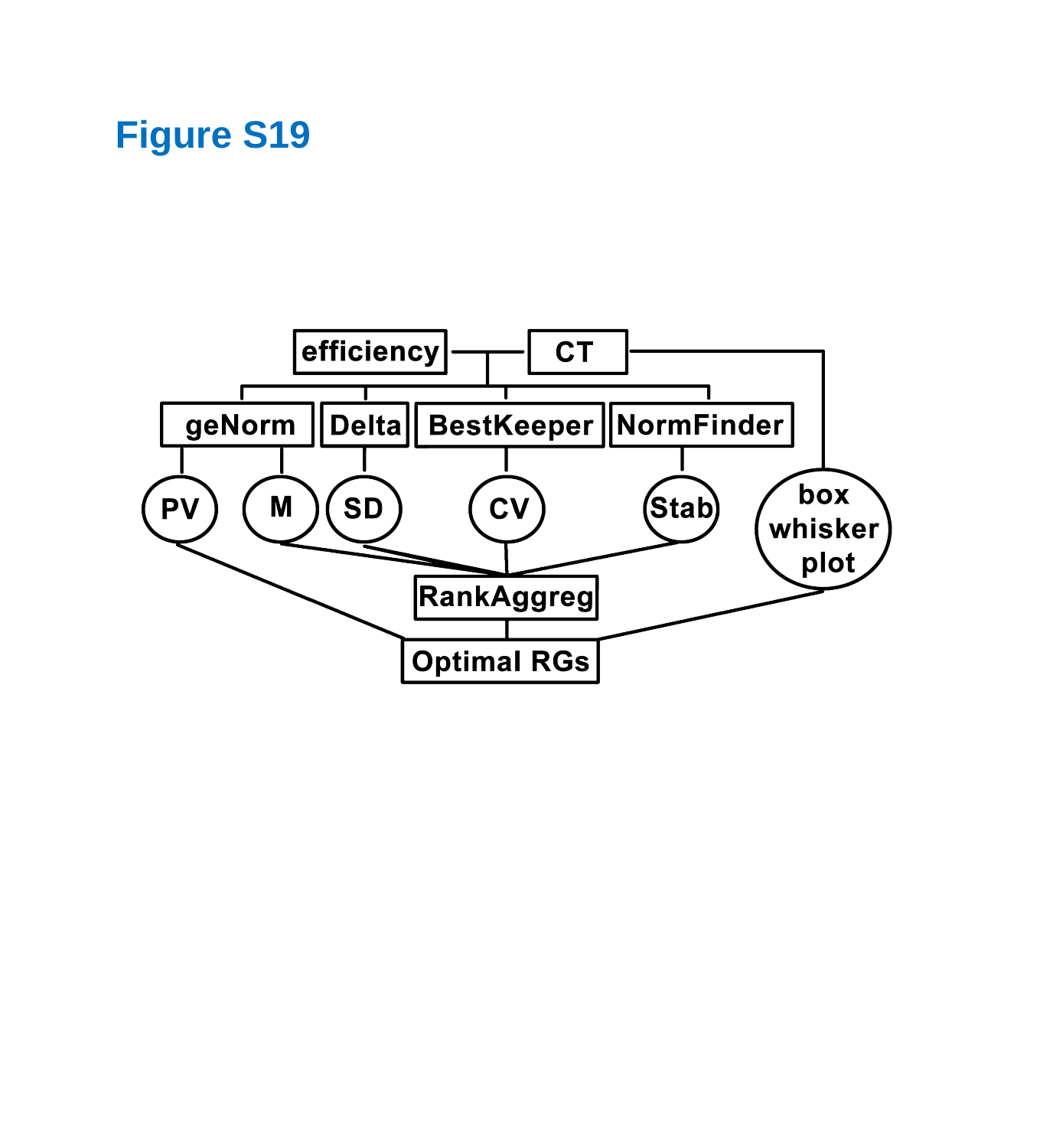

Figure S19

## Slide 20
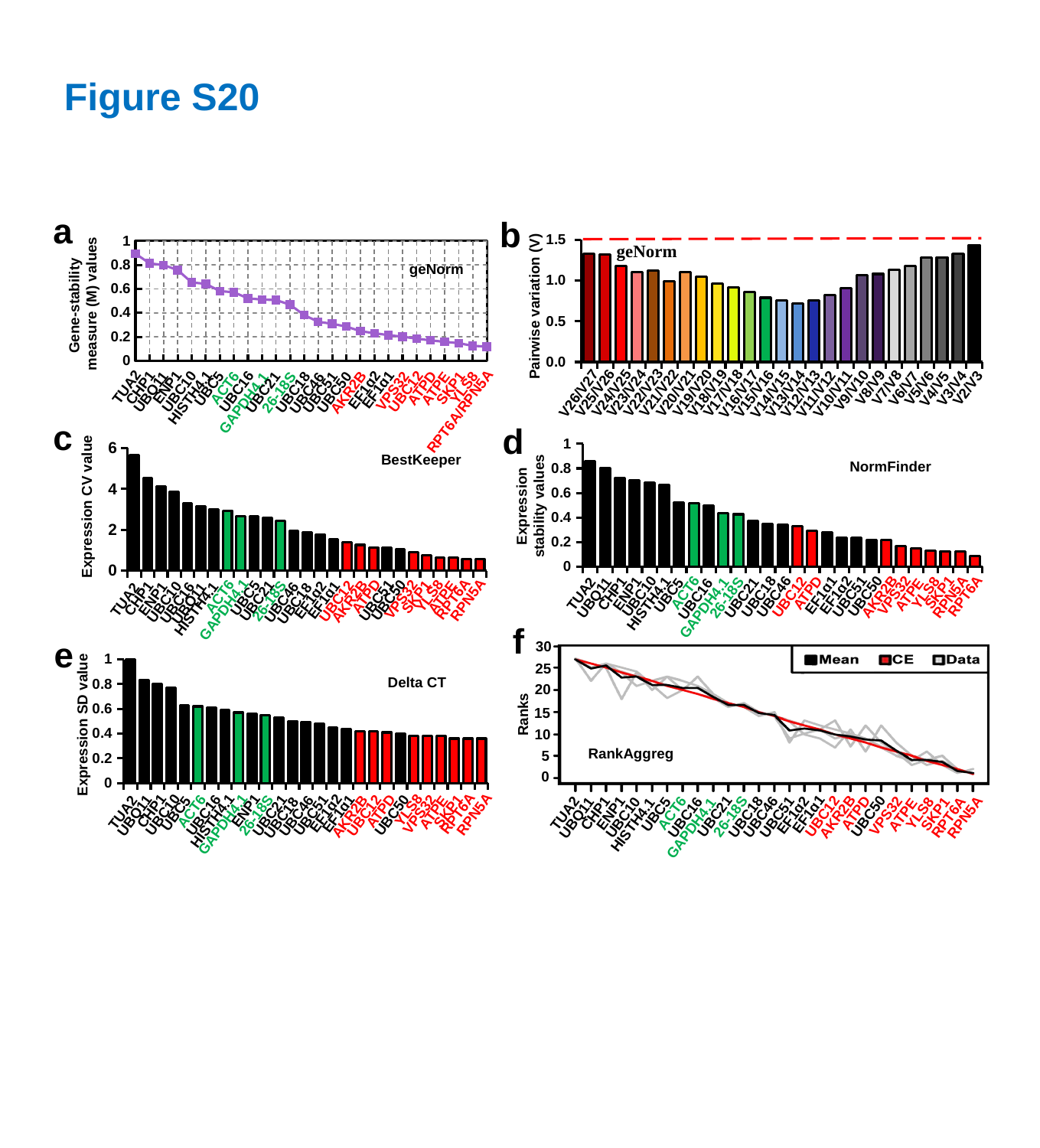

Figure S20
b
geNorm
Pairwise variation (V)
V8/V9
V7/V8
V5/V6
V2/V3
V3/V4
V6/V7
V4/V5
V9/V10
V22/V23
V19/V20
V20/V21
V21/V22
V18/V19
V17/V18
V15/V16
V12/V13
V10/V11
V13/V14
V11/V12
V14/V15
V16/V17
V25/V26
V24/V25
V23/V24
V26/V27
### Chart
| Category | |
|---|---|
| V26/V27 | 1.33 |
| V25/V26 | 1.32 |
| V24/V25 | 1.18 |
| V23/V24 | 1.1 |
| V22/V23 | 1.12 |
| V21/V22 | 0.99 |
| V20/V21 | 1.1 |
| V19/V20 | 1.05 |
| V18/V19 | 0.96 |
| V17/V18 | 0.92 |
| V16/V17 | 0.86 |
| V15/V16 | 0.79 |
| V14/V15 | 0.76 |
| V13/V14 | 0.72 |
| V12/V13 | 0.76 |
| V11/V12 | 0.82 |
| V10/V11 | 0.91 |
| V9/V10 | 1.07 |
| V8/V9 | 1.08 |
| V7/V8 | 1.13 |
| V6/V7 | 1.18 |
| V5/V6 | 1.28 |
| V4/V5 | 1.285 |
| V3/V4 | 1.328 |
| V2/V3 | 1.437 |
### Chart
| Category | |
|---|---|
| TUA2 | 0.894 |
| CHP1 | 0.813 |
| UBQ11 | 0.799 |
| ENP1 | 0.757 |
| UBC5 | 0.6533 |
| HISTH4.1 | 0.6425 |
| UBC10 | 0.5815 |
| ACT6 | 0.572 |
| UBC16 | 0.521 |
| GAPDH4.1 | 0.5111 |
| UBC21 | 0.5073 |
| 18S | 0.468 |
| UBC18 | 0.3812 |
| UBC46 | 0.326 |
| UBC51 | 0.308 |
| UBC50 | 0.286 |
| AKR2B | 0.247 |
| EF1ɑ2 | 0.23 |
| EF1ɑ1 | 0.214 |
| VPS32 | 0.201 |
| UBC12 | 0.185 |
| ATPD | 0.17 |
| ATPE1 | 0.158 |
| RPN5A | 0.147 |
| YLS8 | 0.125 |
| RPT6A_x000d_/SKP1 | 0.118 |a
geNorm
Gene-stability
measure (M) values
TUA2
UBC21
YLS8
SKP1
ACT6
ATPD
ATPE
UBC5
EF1ɑ1
EF1ɑ2
VPS32
UBC10
UBC12
UBC16
26-18S
UBC51
UBC18
UBC50
UBC46
AKR2B
RPT6A/RPN5A
GAPDH4.1
ENP1
CHP1
UBQ11
HISTH4.1
c
RPT6A
TUA2
YLS8
UBC18
ACT6
ATPD
ATPE
UBC5
EF1ɑ2
EF1ɑ1
VPS32
UBC50
RPN5A
UBC51
26-18S
UBC21
UBC12
UBC10
UBC46
AKR2B
UBC16
GAPDH4.1
SKP1
BestKeeper
Expression CV value
ENP1
CHP1
UBQ11
HISTH4.1
### Chart
| Category | |
|---|---|
| TUA2 | 0.861 |
| UBQ11 | 0.8 |
| CHP1 | 0.724 |
| ENP1 | 0.705 |
| UBC10 | 0.684 |
| HISTH4.1 | 0.668 |
| UBC5 | 0.52 |
| ACT6 | 0.519 |
| UBC16 | 0.498 |
| GAPDH4.1 | 0.435 |
| 26-16S | 0.426 |
| UBC21 | 0.374 |
| UBC18 | 0.35 |
| UBC46 | 0.34 |
| UBC12 | 0.332 |
| ATP5 | 0.295 |
| EF1ɑ1 | 0.278 |
| EF1ɑ2 | 0.237 |
| UBC51 | 0.235 |
| UBC50 | 0.218 |
| AKR2B | 0.217 |
| SKP1 | 0.165 |
| ATPE1 | 0.147 |
| RPN5A | 0.128 |
| VPS32 | 0.125 |
| YLS8 | 0.125 |
| RPT6A_x000d_ | 0.084 |d
NormFinder
RPT6A
UBQ11
UBC21
YLS8
ACT6
ATPD
ATPE
UBC5
EF1ɑ2
EF1ɑ1
VPS32
UBC10
UBC50
UBC51
UBC18
UBC46
UBC12
26-18S
AKR2B
RPN5A
UBC16
GAPDH4.1
SKP1
 Expression
stability values
ENP1
CHP1
TUA2
HISTH4.1
### Chart
| Category | |
|---|---|
| TUA2 | 5.67 |
| CHP1 | 4.55 |
| ENP1 | 4.13 |
| UBC10 | 3.8500000000000005 |
| UBC16 | 3.29 |
| UBQ11 | 3.15 |
| HISTH4.1 | 3.01 |
| ACT6 | 2.94 |
| GAPDH4.1 | 2.66 |
| UBC5 | 2.66 |
| UBC21 | 2.59 |
| 18S | 2.4499999999999997 |
| UBC46 | 1.9600000000000002 |
| UBC18 | 1.8900000000000001 |
| EF1ɑ2 | 1.75 |
| EF1ɑ1 | 1.54 |
| UBC12 | 1.4000000000000001 |
| AKR2B | 1.26 |
| ATP5 | 1.12 |
| UBC51 | 1.12 |
| UBC50 | 1.05 |
| VPS32 | 0.91 |
| SKP1 | 0.77 |
| YLS8 | 0.63 |
| ATPE | 0.63 |
| RPT6A_x000d_ | 0.56 |
| RPN5A | 0.56 |
### Chart
| Category | |
|---|---|
| TUA2 | 1.02 |
| UBQ11 | 0.83 |
| CHP1 | 0.8 |
| UBC10 | 0.77 |
| UBC5 | 0.63 |
| ACT6 | 0.62 |
| UBC16 | 0.61 |
| HISTH4.1 | 0.59 |
| GAPDH4.1 | 0.57 |
| ENP1 | 0.56 |
| 18S | 0.55 |
| UBC21 | 0.53 |
| UBC18 | 0.5 |
| UBC46 | 0.49 |
| UBC51 | 0.48 |
| EF1ɑ2 | 0.45 |
| EF1ɑ1 | 0.44 |
| AKR2B | 0.42 |
| UBC12 | 0.42 |
| ATPD | 0.41 |
| UBC50 | 0.4 |
| YLS8 | 0.38 |
| VPS32 | 0.38 |
| ATPE1 | 0.38 |
| RPT6A_x000d_ | 0.36 |
| RPN5A | 0.36 |
| SKP1 | 0.36 |e
Delta CT
RPT6A
TUA2
YLS8
UBC18
ACT6
ATPD
ATPE
UBC5
EF1ɑ2
EF1ɑ1
VPS32
UBC10
RPN5A
UBC16
UBC51
UBC50
26-18S
UBC12
UBC21
UBC46
AKR2B
GAPDH4.1
SKP1
 Expression SD value
ENP1
CHP1
UBQ11
HISTH4.1
f
30
25
20
Ranks
15
10
ACT6
YLS8
ATPD
SKP1
ATPE
UBC5
EF1ɑ1
EF1ɑ2
VPS32
26-18S
UBC50
UBC12
UBC21
UBC18
UBC46
UBC51
UBC16
RPT6A
AKR2B
RPN5A
HISTH4.1
GAPDH4.1
RankAggreg
5
0
TUA2
ENP1
CHP1
UBC10
UBQ11

## Slide 21
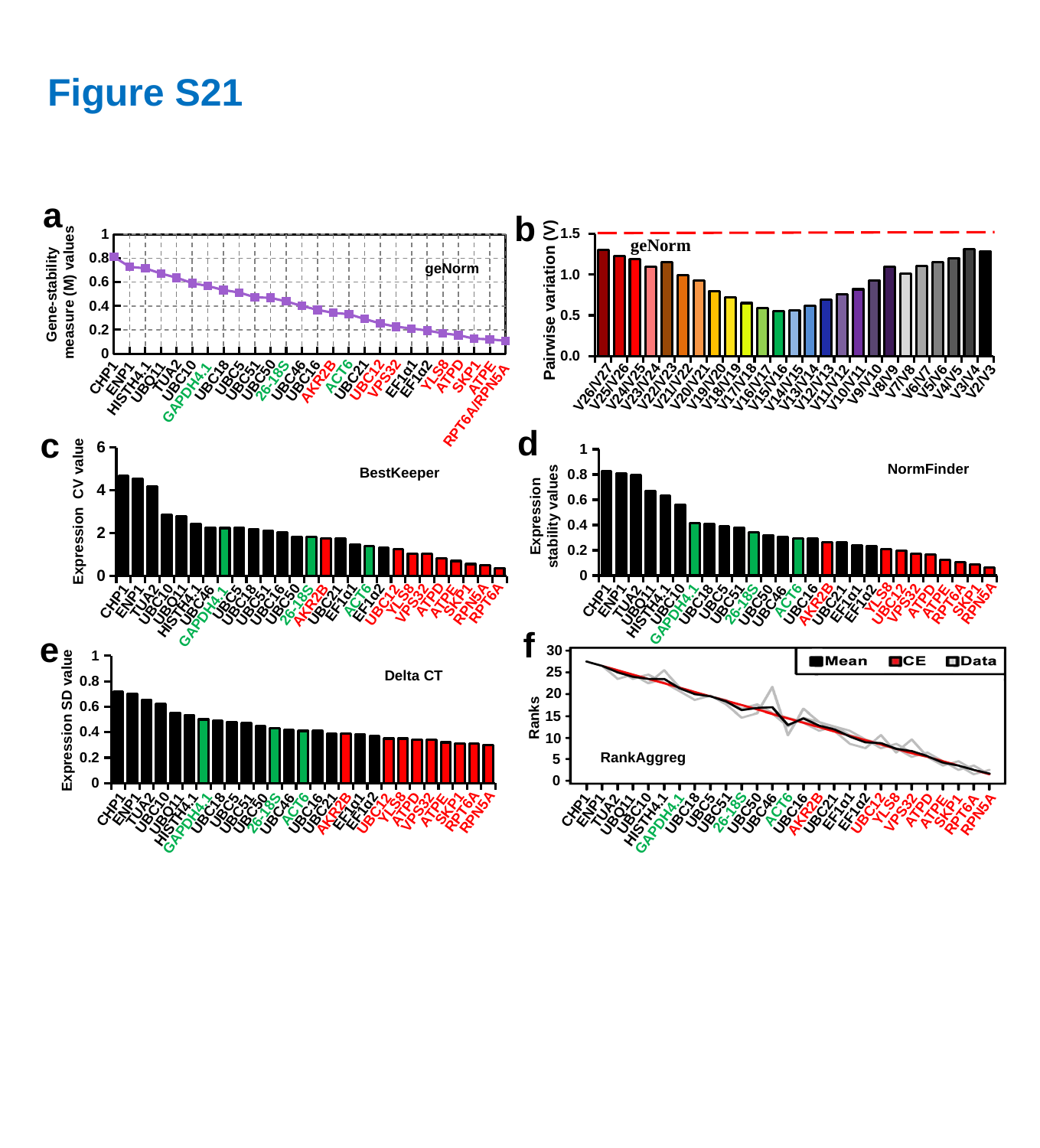

Figure S21
b
geNorm
Pairwise variation (V)
V8/V9
V7/V8
V5/V6
V2/V3
V3/V4
V6/V7
V4/V5
V9/V10
V22/V23
V19/V20
V20/V21
V21/V22
V18/V19
V17/V18
V15/V16
V12/V13
V10/V11
V13/V14
V11/V12
V14/V15
V16/V17
V25/V26
V24/V25
V23/V24
V26/V27
### Chart
| Category | |
|---|---|
| V26/V27 | 1.3 |
| V25/V26 | 1.23 |
| V24/V25 | 1.19 |
| V23/V24 | 1.1 |
| V22/V23 | 1.15 |
| V21/V22 | 0.99 |
| V20/V21 | 0.93 |
| V19/V20 | 0.8 |
| V18/V19 | 0.72 |
| V17/V18 | 0.65 |
| V16/V17 | 0.59 |
| V15/V16 | 0.55 |
| V14/V15 | 0.56 |
| V13/V14 | 0.62 |
| V12/V13 | 0.69 |
| V11/V12 | 0.76 |
| V10/V11 | 0.82 |
| V9/V10 | 0.93 |
| V8/V9 | 1.1 |
| V7/V8 | 1.01 |
| V6/V7 | 1.11 |
| V5/V6 | 1.15 |
| V4/V5 | 1.2 |
| V3/V4 | 1.31 |
| V2/V3 | 1.28 |
### Chart
| Category | |
|---|---|
| CHP1 | 0.811 |
| ENP1 | 0.728 |
| HISTH4.1 | 0.715 |
| UBQ11 | 0.672 |
| TUA2 | 0.637 |
| UBC10 | 0.591 |
| GAPDH4.1 | 0.567 |
| UBC18 | 0.5333 |
| UBC5 | 0.5125 |
| UBC51 | 0.473 |
| UBC50 | 0.468 |
| 18S | 0.4385 |
| UBC46 | 0.3997 |
| UBC16 | 0.3672 |
| AKR2B | 0.342 |
| ACT6 | 0.331 |
| UBC21 | 0.291 |
| UBC12 | 0.2535 |
| VPS32 | 0.226 |
| EF1ɑ1 | 0.211 |
| EF1ɑ2 | 0.194 |
| YLS8 | 0.171 |
| ATPD | 0.155 |
| RPN5A | 0.126 |
| ATPE1 | 0.12 |
| RPT6A_x000d_/SKP1 | 0.108 |a
geNorm
Gene-stability
measure (M) values
TUA2
UBC21
YLS8
ACT6
ATPD
SKP1
ATPE
UBC5
EF1ɑ1
EF1ɑ2
VPS32
UBC12
UBC50
UBC18
26-18S
UBC46
UBC51
UBC10
UBC16
AKR2B
GAPDH4.1
RPT6A/RPN5A
CHP1
ENP1
UBQ11
HISTH4.1
c
BestKeeper
RPT6A
TUA2
UBC18
YLS8
ATPD
ACT6
ATPE
UBC5
EF1ɑ1
EF1ɑ2
VPS32
UBC10
RPN5A
UBC50
26-18S
UBC21
UBC51
UBC12
UBC16
UBC46
AKR2B
GAPDH4.1
SKP1
Expression CV value
ENP1
CHP1
UBQ11
HISTH4.1
### Chart
| Category | |
|---|---|
| CHP1 | 0.828 |
| ENP1 | 0.81 |
| TUA2 | 0.794 |
| UBQ11 | 0.672 |
| HISTH4.1 | 0.631 |
| UBC10 | 0.558 |
| GAPDH4.1 | 0.414 |
| UBC18 | 0.407 |
| UBC5 | 0.389 |
| UBC51 | 0.376 |
| 26-16S | 0.34 |
| UBC50 | 0.315 |
| UBC46 | 0.307 |
| ACT6 | 0.293 |
| UBC16 | 0.293 |
| AKR2B | 0.263 |
| UBC21 | 0.262 |
| EF1ɑ1 | 0.24 |
| EF1ɑ2 | 0.234 |
| YLS8 | 0.207 |
| UBC12 | 0.195 |
| VPS32 | 0.174 |
| ATP5 | 0.169 |
| ATPE1 | 0.123 |
| RPT6A_x000d_ | 0.105 |
| SKP1 | 0.088 |
| RPN5A | 0.066 |d
NormFinder
RPT6A
TUA2
UBC21
YLS8
ACT6
ATPD
ATPE
UBC5
EF1ɑ1
EF1ɑ2
VPS32
RPN5A
UBC51
26-18S
UBC10
UBC16
UBC12
UBC18
UBC50
AKR2B
UBC46
GAPDH4.1
SKP1
 Expression
stability values
ENP1
CHP1
UBQ11
HISTH4.1
### Chart
| Category | |
|---|---|
| CHP1 | 4.69 |
| ENP1 | 4.55 |
| TUA2 | 4.2 |
| UBC10 | 2.8699999999999997 |
| UBQ11 | 2.8000000000000003 |
| HISTH4.1 | 2.4499999999999997 |
| UBC46 | 2.24 |
| GAPDH4.1 | 2.24 |
| UBC5 | 2.24 |
| UBC18 | 2.17 |
| UBC51 | 2.1 |
| UBC16 | 2.03 |
| UBC50 | 1.82 |
| 18S | 1.82 |
| AKR2B | 1.75 |
| UBC21 | 1.75 |
| EF1ɑ1 | 1.47 |
| ACT6 | 1.4000000000000001 |
| EF1ɑ2 | 1.33 |
| UBC12 | 1.26 |
| YLS8 | 1.05 |
| VPS32 | 1.05 |
| ATP5 | 0.84 |
| ATPE | 0.7000000000000001 |
| SKP1 | 0.56 |
| RPN5A | 0.49000000000000005 |
| RPT6A_x000d_ | 0.35000000000000003 |
### Chart
| Category | |
|---|---|
| CHP1 | 0.72 |
| ENP1 | 0.7 |
| TUA2 | 0.65 |
| UBC10 | 0.62 |
| UBQ11 | 0.55 |
| HISTH4.1 | 0.53 |
| GAPDH4.1 | 0.5 |
| UBC18 | 0.49 |
| UBC5 | 0.48 |
| UBC51 | 0.47 |
| UBC50 | 0.45 |
| 18S | 0.43 |
| UBC46 | 0.42 |
| ACT6 | 0.41 |
| UBC16 | 0.41 |
| UBC21 | 0.39 |
| AKR2B | 0.39 |
| EF1ɑ1 | 0.38 |
| EF1ɑ2 | 0.37 |
| UBC12 | 0.35 |
| YLS8 | 0.35 |
| ATPD | 0.34 |
| VPS32 | 0.34 |
| ATPE1 | 0.32 |
| SKP1 | 0.31 |
| RPT6A_x000d_ | 0.31 |
| RPN5A | 0.3 |e
Delta CT
RPT6A
UBC18
YLS8
ACT6
ATPD
ATPE
UBC5
EF1ɑ1
EF1ɑ2
VPS32
TUA2
UBC10
RPN5A
26-18S
UBC21
UBC51
UBC50
UBC16
UBC12
UBC46
AKR2B
GAPDH4.1
SKP1
 Expression SD value
UBQ11
ENP1
CHP1
HISTH4.1
f
30
25
20
Ranks
15
10
ACT6
YLS8
ATPD
SKP1
UBC5
ATPE
EF1ɑ1
EF1ɑ2
VPS32
26-18S
UBC51
UBC18
UBC50
UBC12
UBC46
UBC21
UBC16
RPT6A
AKR2B
RPN5A
HISTH4.1
GAPDH4.1
ENP1
CHP1
TUA2
UBC10
UBQ11
RankAggreg
5
0
